# Influences on the Implementation of Mobile Learning for Medical and Nursing Education: Qualitative Systematic Review by the Digital Health Education Collaboration

**DOI:** 10.2196/12895

**Published:** 2019-02-28

**Authors:** Priya Lall, Rebecca Rees, Gloria Chun Yi Law, Gerard Dunleavy, Živa Cotič, Josip Car

**Affiliations:** 1 School of Geography Queen Mary University of London London United Kingdom; 2 Evidence for Policy and Practice Information and Co-ordinating Centre, Social Science Research Unit, Department of Social Science University College London Institute of Education University College London London United Kingdom; 3 Centre of Population Health Sciences Lee Kong Chian School of Medicine Nanyang Technological University Singapore Singapore; 4 Faculty of Social Sciences University of Ljubljana Ljubljana Slovenia; 5 Faculty of Medicine School of Public Health Imperial College London London United Kingdom

**Keywords:** medical education, nursing education, distance education, qualitative research, systematic review

## Abstract

**Background:**

In the past 5 decades, digital education has increasingly been used in health professional education. Mobile learning (mLearning), an emerging form of educational technology using mobile devices, has been used to supplement learning outcomes through enabling conversations, sharing information and knowledge with other learners, and aiding support from peers and instructors regardless of geographic distance.

**Objective:**

This review aimed to synthesize findings from qualitative or mixed-methods studies to provide insight into factors facilitating or hindering implementation of mLearning strategies for medical and nursing education.

**Methods:**

A systematic search was conducted across a range of databases. Studies with the following criteria were selected: examined mLearning in medical and nursing education, employed a mixed-methods or qualitative approach, and published in English after 1994. Findings were synthesized using a framework approach.

**Results:**

A total of 1946 citations were screened, resulting in 47 studies being selected for inclusion. Most studies evaluated pilot mLearning interventions. The synthesis identified views on valued aspects of mobile devices in terms of efficiency and personalization but concerns over vigilance and poor device functionality; emphasis on the social aspects of technology, especially in a clinical setting; the value of interaction learning for clinical practice; mLearning as a process, including learning how to use a device; and the importance of institutional infrastructure and policies.

**Conclusions:**

The portability of mobile devices can enable interactions between learners and educational material, fellow learners, and educators in the health professions. However, devices need to be incorporated institutionally, and learners and educators need additional support to fully comprehend device or app functions. The strategic support of mLearning is likely to require procedural guidance for practice settings and device training and maintenance services on campus.

## Introduction

### Background

In the past 5 decades, digital education has increasingly been used in health professional education, and technological advances have produced various forms of digital education modalities such as computer-based simulations, virtual patients, and internet-based courses and interactive contents [1,2]. Adoption of these digital education modalities in health professional education is rapidly expanding before the establishment of a robust evidence base for consideration of multiple dimensions and outcomes [3,4]. A noteworthy trend within digital education is mobile learning (mLearning), which can be defined as follows [5]:

...consuming, interacting with or creating information, mediated through a compact digital portable device that the individual carries on a regular basis, has reliable connectivity, and fits in a pocket or purse.

This is enabled by a growth of capabilities in mobile devices (eg, smartphones) and the convenience they offer, such as omnipresent usability and accessibility to the internet, while mobile. Approximately 1.1 billion people living in rural areas [6] and 73% of the total world population [7] are now covered by mobile broadband.

Mobile devices can offer a variety of functions and be used across contexts. For instance, mLearning can provide access to educational content and information in daily clinical practice [8-10]; enable conversations and the sharing of information and knowledge with other learners; and elicit support from peers and instructors regardless of geographic distance [8-10]. Handheld computers can be used to keep track of students’ skill development and progress in assignments [11]; promote self-directed and self-regulated learning [12,13]; display audio-visual information relating to a specific place, scene, or situation; and aid situated learning [10].

Evaluations of the effects of digital education and specifically mLearning as a whole raise more questions than they answer. For example, a meta-analysis by Free et al [14] included 7 randomized controlled trials and investigated the educational outcomes associated with the use of personal digital assistants (PDAs) and portable media players in medical and nursing education. The studies incorporated into the systematic review examined the effectiveness of mLearning in improving knowledge and attitudes; however, the meta-analysis showed no clear evidence of benefit. There are many factors influencing the effectiveness of digital education and mLearning interventions that warrant closer investigation. The implementation of digital education can be influenced by characteristics of the educational intervention, problems addressed by the intervention, features of the health system, the adopting system, and other contextual factors [15]. However, no review has been identified which examines systematically the factors influencing the use of mLearning interventions for health professional education.

Our review considered the broad issue of *implementation* of mLearning. This is important because mLearning is a relatively new area of development compared with other forms of digital education. We are in the early stages of learning what happens and what might be helpful when mLearning is introduced into real-world settings. Having a systematic and in-depth exploration of the range of potential *barriers to and facilitators* *of* mLearning in health professional education should deepen the understanding of the topic and allow insights to be obtained for effective implementation and positive outcomes. It is also important to understand mLearning in terms of the underlying assumptions about teaching and learning (ie, pedagogy and andragogy) of different approaches, to maximize the potential richness of the learning process in mobile environments and enable teachers to plan for optimal learning [16].

**Figure 1:**
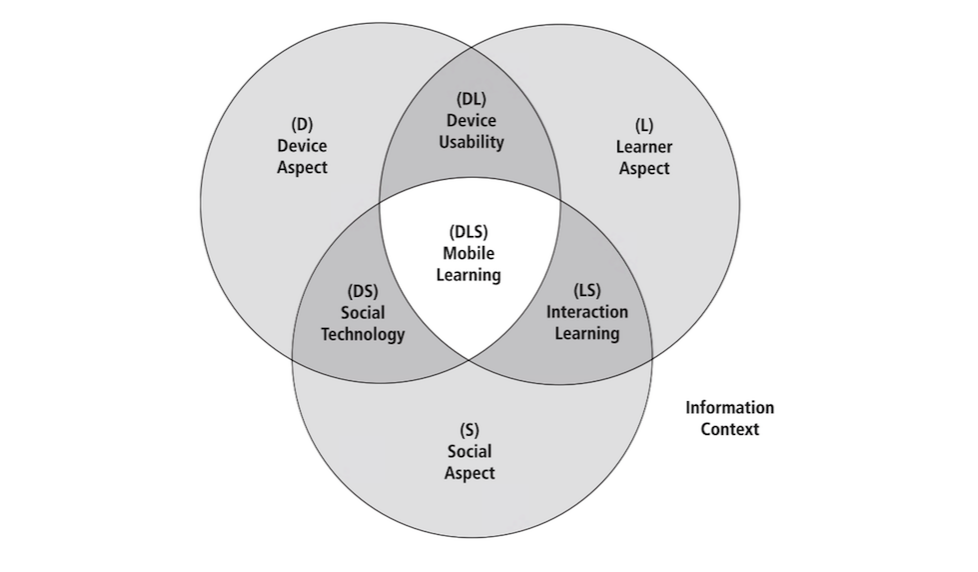
Framework for the Rational Analysis of Mobile Education (FRAME) model.

Koole’s Framework for the Rational Analysis of Mobile Education (FRAME) model guides the qualitative synthesis for this study (see Figure 1) [17]. This model considers how features of mobile technology, along with learner capacities and social interaction, influence learning processes occurring in an information context. Within the FRAME model, mLearning is conceived as the convergence of the following aspects: (1) device, signifying functional characteristics of a mobile device, for example, processor speed; (2) learner, accounting for individuals’ cognitive abilities and learning styles; and (3) social, referring to elements of social interaction and culture-influencing learning processes.

In terms of interactions between these aspects, first, *Device Usability* is thought of as containing aspects belonging to the device and learner and describes how an individual relates to the device. For example, learners can express satisfaction with a particular device because of its esthetic qualities. Second, *Social Technology* covers the intersection between device and social aspects and accounts for how mobile devices enable connection between multiple interfacing individuals and systems, such as the use of collaborative tools. Finally, *Interaction Learning* spans the intersection between learner and social aspects, describing how the learner interacts with other individuals. For instance, a mobile device could enable interaction between a learner and their instructor on long-distance educational courses. The culmination of all 3 aspects is envisioned as the eventual process of mLearning.

### Objectives

This study aimed to synthesize insights from empirical research using qualitative and mixed methods on mLearning implementation in medical and nursing education. Our study employed systematic methods to identify, appraise, and synthesize qualitative findings from studies to explore mLearning strategies for medical and nursing education. These studies can allow us to better understand the nature of material and sociocultural influences (eg, cultural norms) and causal pathways [18] to delineate a more complete picture of the phenomenon under study [19]. Qualitative findings from existing studies are used to uncover the perspectives of learners and other key actors with experience of mLearning strategies. Particular attention is paid to perceptions of implementation processes. The broad research question for this review was as follows: What are the views of educators, learners, and other key actors with experience of mLearning in medical and nursing education about perceived factors which facilitate/enhance or hinder its implementation?

## Methods

### Protocol

A protocol was developed so as to establish the review’s scope and methods before evaluating existing literature. This was registered with PROSPERO, the international prospective register of systematic reviews (record number CRD42016035411 Multimedia Appendix 1) [20].

### Inclusion Criteria

Studies were included if they examined medical and/or nursing students’ (or their educators’) perspectives on or experiences of mLearning. They also needed to be written in English, to involve some form of qualitative data collection or analysis (eg, focus group interviews), to collect data from learners in medical or nursing education who were involved in mLearning as defined by Wexler et al [5], and to be published after 1994.

### Identifying Relevant Studies

We conducted a comprehensive search that combined terms for the concepts of digital technology, education, and health professionals. This search was conducted in February 2015 and was repeated in March 2017 on 8 electronic databases (MEDLINE, EMBASE, Cochrane Library, PsycINFO, ERIC, CINAHL, Web of Science, and International Clinical Trials Platform). Databases were searched from and including the year 1995 to March 2017 (see Multimedia Appendix 2).

**Figure 2:**
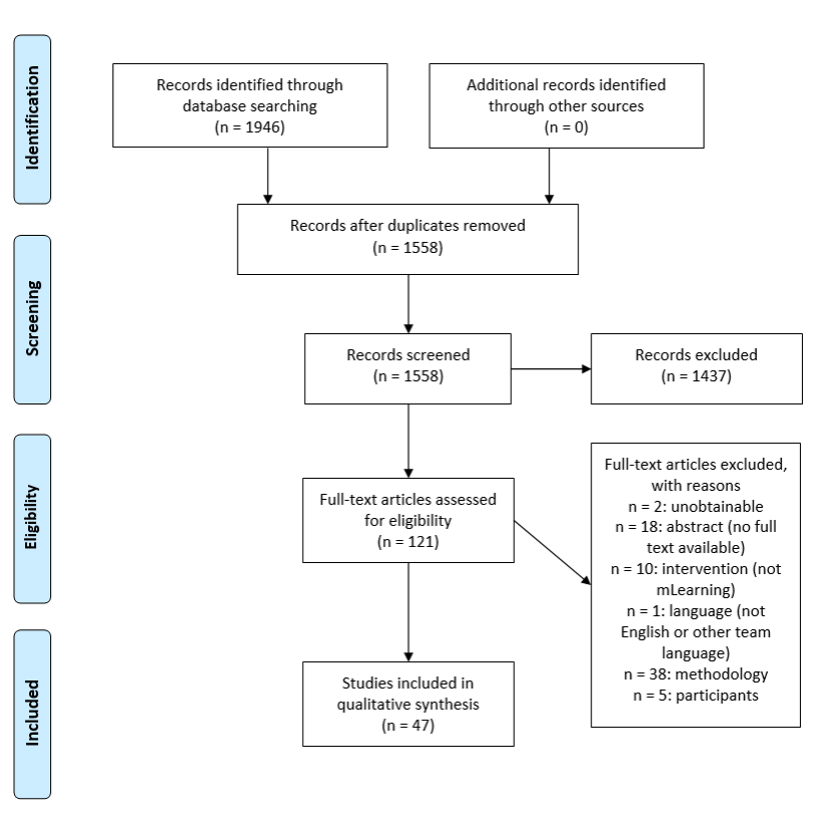
Preferred Reporting Items for Systematic Reviews and Meta-Analyses chart.

All references identified were uploaded to the specialist systematic review software EPPI-Reviewer 4 (University College London) [21], and data deduplication was performed within this program. A second phase of searches was then run to identify qualitative studies of mLearning using the EPPI-Reviewer search function. These searches looked for items that had terms related to qualitative research and to mLearning (see Multimedia Appendix 2). The resulting set of references was assessed against our predefined inclusion and exclusion criteria. The criteria were developed by all authors and piloted by 4 authors (GD, GL, PL, and ZC) on a randomly selected sample of studies. The pilot was completed after there was a high level of agreement (over 90%) on the selection of studies between all 4 authors. Abstracts and full texts were each independently screened by 2 of these same 4 authors. In cases where there were difficulties reaching consensus on inclusion of a particular text, a third provided the deciding judgment. Figure 2 shows the Preferred Reporting Items for Systematic Reviews and Meta-Analyses flowchart for the study.

### Describing Studies and Appraising Their Quality

Features of the included studies were described according to the following characteristics: (1) aim; (2) sample characteristics (ie, size, country, and study population); (3) type of mLearning device and apps used; (4) type of study (ie, study of an intervention or inquiry into an existing phenomenon); (5) type of mLearning (eg, reference repository); and (6) study design, such as the type of data collection and sampling approach.

Quality of the final set of studies was assessed using a tool used in previous studies [22,23], where the quality of each study is rated using a total of 7 questions set within 2 dimensions—trustworthiness and usefulness of the findings. The first dimension captures the degree to which the methods were used to ensure rigor; the second, for the purposes of this review, focused on the complexity of analysis of the mLearning strategy. Furthermore, 2 reviewers independently rated each study (high, medium, or low for each dimension) and then compared judgments before coming to a consensus. Studies that met the inclusion criteria were included in the review regardless of the study quality, with ratings described alongside other characteristics of the papers.

### Analysis and Synthesis

Themes were identified using a framework analysis approach in which data are reduced through the development of a matrix, comparing categories of data or cases, and a synthesis is developed using an initial theoretical framework. Data were analyzed according to phases of analysis identified by Pope et al [24], starting with 3 authors (PL, GL, and GD) familiarizing themselves with the selected studies. These authors coded selected studies according to the FRAME model. The information gathered from coded text was distilled into a chart containing summaries of the themes. After the search update, 3 authors (PL, GL, and GD) undertook the same process, further modifying the themes and subthemes. When findings were found in texts which addressed an area not covered in the initial framework, the framework themes were added to or modified accordingly. Two authors (PL and GL) wrote a narrative to describe and illustrate the themes and their relationships. In addition, 1 author (RR) then familiarized herself with all the studies and worked further with the lead author on the synthesis narrative and themes, using study texts to check and further incorporate references to individual studies.

## Results

### Research Questions Being Addressed and Quality of Studies

The 47 studies in this synthesis [2,10,12,25-68] varied according to study context and participant types, the research questions being addressed, types of mLearning strategies used, and aspects of study design (see Multimedia Appendix 3). A total of 37 studies were conducted in high-income countries [2,10,12,25,27-42,45-50,52,56-58,60-62,64-66], for example, the United Kingdom (n=9) [10,12,29,31,34,40,41,61,66], with the remainder set in lower-income settings [26,43,44,51, 53-55,59,63,67,68], including India [44,55,59], South Africa [53,63], Botswana [26,68], and Rwanda [67].

Studies mainly sought views of learners, but some also included educators (n=11) [26,30,39,45-48,53,61,64,65] or focused solely on educators (n=4) [30,47,48,61]. Most studies focused solely on the experience of medical staff or students (n=24) [10,12,25,27,28,30,31,33,36,40,42-45,49,51,54-56,58,60,62,67,68] whereas a smaller number of studies sampled either solely from nursing staff and students (n=19) [2,29,32,35,37,38,46-48,50, 52,53,57,59,61,63-66] or from a mixture of both doctors and nurses (n=4) [26,34,39,41]. Students were at different stages of education and so were learning in different settings. A small number of studies looked at device use aimed at supporting learning in undergraduate lecture, seminar, or laboratory environments (n=5) [29,38,43,61,63]. In all, 7 studies sought views on mLearning for the further professional and/or academic development of fully qualified doctors or nurses [31,35,52,55,57,67]. Most studies, however, sought the views of nursing and medical students, or educators, about mLearning during various clinical placements before health professional registration.

The purpose of the majority of studies was to evaluate pilot mLearning approaches (n=32) [10,12,25-29,31-33,37-44, 46,47,49,55,58-66,68] that were implemented in medical and nursing contexts. Furthermore, 2 of these evaluations examined the provision of mobile hardware without describing specific software arrangements [33,60]. In a further 9 evaluations, mobile devices had been designed primarily to be reference repositories [10,12,28,31, 32,37,41,49,68], for example, students were loaned a PDA with preloaded medical texts by their institutions [28]. In a further 8 evaluations, devices were aimed at supporting learning through use of a suite of recommended apps or software [25,27,39,40,47,61,62,65]. In 3 of these 8, the studies focused in particular on students’ use of electronic logs or ePortfolios to reflect on and/or evaluate their experiences or learning [27,39,40]. The remaining evaluations examined a variety of specific mLearning strategies, including the use of multimodal techniques (eg, those using videos of clinical skills, whiteboards, and presentation software) for group or individual activities (n=5) [29,42,55,59,64], augmented reality (n=2) [38,43], messaging services (n=4) [26,44,46,66], a social media–enabled discussion group (n=1) [63], and a mobile app to prompt specific clinical behaviors [58].

Of the remaining studies, a further 4 explored the co-design of, or needs for, specific future mLearning interventions [45,50,51,67]. A final set of 11 studies were not conducted with the purpose of designing or evaluating a specific intervention. Instead, these studies explored students’ experiences of using mobile devices to enable their own learning in the absence of an institutionally planned mLearning initiative [2,30,34-36,48,52-54,56,57].

Studies predominantly employed a mixed-methods research design (n=33). These studies used one or a mix of qualitative data collection methods, such as focus group discussions (n=13) [12,26,27,33,37-40,55,59,60,65,68], group or individual interviews (n=15) [25,34-36,41-44,49,51,52,58,64,66,67], and analysis of textual survey responses (n=9) [12,27,29,32, 34,36,40,46,67]. A smaller number of studies used only qualitative methods (n=14) [28,30,31,45,47,48,50,53,54, 56,57,61-63], which included focus group discussions (n=6) [28,47,48,50,54,63], group or individual interviews (n=7) [30,45,53, 56,57,61,63], textual reflection or journals (n=4) [31,47,61,63], and participant observation [57].

Multimedia Appendix 4 displays the quality appraisal of studies included in this synthesis in terms of quality. Ten studies were judged to have highly reliable findings [12,28,47,51-54, 58,59,62] whereas 12 were deemed to be highly useful for this review [12,29,31,39,49,53,54,56,57,62,63,68]. Only 4 studies were considered both highly reliable and useful [12,53,54,62]. The ratings for each study are listed in Multimedia Appendix 3 and Multimedia Appendix 4.

### Substantive Findings

The narrative below presents an overview of study participants’ views of mLearning organized under the spaces in which the device, learner, and their social setting interact (device usability, social technology, interaction learning, mLearning processes and implementation in clinical contexts). Table 1 provides illustrative quotations. The full synthesis narrative, which includes citations to the studies that support each theme, is available as Multimedia Appendix 5.

Analysis revealed that the progress of mLearning strategies in medical and nursing education often appeared to be shaped by processes that were out of the hands of learners and their teaching staff. Instead, issues raised sometimes related to other actors in the institutional contexts in which learning was taking place and the implementation of policies within these learning settings. An additional factor shaping the operation of mLearning strategies was social norms governing the use of mobile devices in clinical and classroom settings.

**Table 1:** Illustrative quotes according to theme.

FRAME model themes		Quotes (from learners unless otherwise specified)
**Device usability**		
	Portability means efficiency but also vigilance	“Much, much quicker than flicking through the paper version. . . Looking things up in the paper BNF [British National Formulary] for the n-th time on ward rounds puts time pressure on the junior doctor causing stress and increasing risk of errors.” [10, p. 8]
		“You could do that [feedback] in a few minutes on your phone, rather than doing it or on a piece of paper that you lose.” [40, p. 928]
		“Carrying books is a drag, now I’m a ‘lightweight’.” [28, p. 614]
		“The places I feel uncomfortable using [the mobile device] are outside, like in the mall or in a kombi [public transportation], because it’s sort of a big thing, and I think it could attract thieves.” [68, p. 75]
	Fit for purpose hardware, software, and data	“I preferred working on the e-portfolio and entering data via computer as the screen was too small on the PDA to be practical and efﬁcient.” [39, p. 652]
		“The use of the device got me thinking what I actually needed and the sheer fact that a laptop is too large and cumbersome to carry around with you. I wanted something that I could boot up quite instantly and get on the Wi-Fi; go transfer ﬁles and this is ideal.” [62, p. 574]
		“I think [a tablet] would be better than a [smartphone] because if it was an [tablet] you could actually have lectures on there and it would be big enough to read and work on.” [40, p. 928]
	Ownership, personalization, and sense of self	“I can access it [the mobile device] anytime ... and it is mine to use ...” [28, p. 613]
		“I’ve sometimes forgotten my handheld and had the feeling of being naked in a way.” [28, p. 616]
		“It is part of my life now […] a means of contact, a means of learning. You know, people who have phones just learn a lot.” [53, p. 1401]
		“I find I am having more and more problems with exams because I cannot look up easily what I normally look up... everyday on my [smartphone].” [33, p. 134]
**Social technology**		
	Devices can impact care and learning relationships	“Well, it’s not that I don’t use a [PDA], I use it for looking up drugs and things, but I think in a conversation it is kind of awkward to kind of pull it out and break eye contact.” [58, p. 5]
		“Because [the doctors] think that I’m not concentrating with them while using technology, whether it’s [a smartphone or tablet]… I’m writing notes or something, but … in the beginning they didn’t like the fact that I’m using this.” [57, p. 5]
	Devices raise issues of professionalism and practice boundaries	“These days with the younger generation, if you pull out your [tablet or PDA] and you come up with the information, you are seen as competent. You are seen as having the advanced knowledge. If you say 'well just a minute, I have to go find my book' and you are flipping through the book then you are seen as old fashioned and that you aren't as current as you should be.” [35, p. 12]
		“You know someone will say ‘Hey put your phone down’ or ‘Check your message later’ or something and you can’t say ‘Oh I’m actually looking…’ it just looks unprofessional so to be honest I don’t use it when I’m in front of a patient or with the doctors…When we…on an actual round I am very careful not to pull my phone out because it’s still a phone you know so I think the stigma is that you’re then distracted because it’s a phone and it could be…you know if the doctor is talking.” [56, p. 5]
		“I think some doctors have made comments about ‘What are you doing on that, are you texting someone, or playing games’.” [12, p. 6]
	Negotiating the social aspect of mLearning^a^	“As the patient was an elderly gentleman I was slightly apprehensive that he wouldn’t appreciate me using a phone during the consultation however with explanation of my actions he was perfectly content with my use of [the device].” [31, p. 6]
		“When you are dealing with a patient it is easy to access that list and decide on the right medication together. It is also handy when you have a laboratory result and you want to find out what you can do in terms of additional laboratory research.” [25, p. 332]		
**Interaction learning**		
	Facilitated interaction and learning	“The students explained… ‘[We show the picture] to flat mates. This is the case I have seen. [...] The whole batch gets it. [.. .] We proudly show it to the others’.” [54, p. 1160]
		“I liked the fact that it was anonymous, so it gave me the freedom to ask anything without the fear of being criticised without it feeling as if I’m asking a *stupid* question.” [63, p. 5]
		“[names a social media discussion group], I love it. …I’m part of the group… He [the group convenor] asks questions to medical students and gives the correct answers… there are more than 15000 people.” [54, p. 1160]
		“[Describing a social media facilitated student group]… Sometimes you use the group afterwards, after you have managed the patient, to see how you went, where you went wrong, how you did, or sometimes they say I messed up. Then, they give you the reasons, or sometimes they will tell you, oh, well done, but you missed that and that.” [53, p. 1400]
		“[describing peer evaluation of clinical skills via Skype] I have learnt a lot and by students asking me questions. I feel my own knowledge has improved.” [Educator] [51, p. 467]
	Organizing learning using mobile devices	“…sharing information and allocating tasks to different members …it can allow that interaction to happen across distance. … PDAs would help keeping the interaction that coordinate the [problem based learning] process, in tagging people (peers, clinicians and the …faculty)” [Educator]. [45, p. 116]
		Use of the mobile device during downtime, such as skim reading meeting agendas while on the train …was mentioned as 1 of the main benefits of having the portable device (eg, “…instead of having a paper base you can just scroll through the minutes just to remind yourself”). [61, p. 573]
	Reflective learning for clinical practice	“I don’t use my phone immediately. I will write down the things we didn’t know, we nod our heads and then when we leave we’ll sit on our tea break and look them up quickly to make sure we understand or we know what we are talking about.” [56, p. 4]
		“When we are together [in school settings], we share and discuss the photos. Some [conditions] we learn in school take a long time to see [in practice settings]. So, when you witness this condition and you are not together with your colleagues, you take this picture. […] Then you look at the picture and [later] discuss it, if it corresponds with what we have learned.” [53, p. 1400]
		“[written scenario] When teaching is impromptu, conventional multimedia equipment may be either unavailable or inappropriate. … The portability of the Smartphone facilitated teaching anatomy in the context of its clinical application within general surgery. It provided visual stimuli to enrich several ad hoc teaching experiences in a single day.” [Educator] [10, p. 7]
		“A lot of people also discovered that you could use Facebook on it, and also games and stuff … I feel that when you are in the hospital, or actually when you are in the OR, and you are doing something on your iPod, whatever it is, you will be distracted from the process, and it takes longer to react on the things that are happening.” [57, p. 1106]
**mLearning processes**		
	Changes in pedagogy and learning	“In contrast to the previously mentioned statements made by teachers about students’ uncritical and non-reflective use of ICT, the teachers also acknowledged positive changes with respect to the *division of labour*, as indicated in the following statement by a teacher: ‘There has been a dramatic change. We don’t have to teach everything now. It’s not teacher based learning. It is student based learning. We just tell them and guide them. We give them topics. We tell them to look up and search those topics on the internet and we ask them to verify them from the textbooks. If they find something new and interesting they can ask us. The students are helping us. They are stimulating us to study more. It’s a two way conversation. And the students are also contributing’.” [54, p. 1161]
		“The use of the [tablet] allowed for the shared construction of knowledge between the teachers and the students. One comment was ‘I found the immediacy of this learning immensely powerful for my own learning and the student's … able to look together. In fact, one student pulled their [smartphone] and said, ‘I'll race you!’ While another commented, ‘off into the internet to ﬁnd out together!’ to ﬁnd the answer to a clinical question that neither knew the answer to’.” [47, p. 4]
	Learning to mLearn	“I was quite averse to it at first –I was one of the haters... [interviewer: What changed your mind?]… I think it’s actually finding I did use the PDA and it did come in handy several times. It just makes life a bit easier.” [12, p. 7]
		“Actually, I was shown by my daughter at home. […] So I showed my colleagues, yeah.” [53, p. 1401]
		“[talking about not being able to view past assessments on a smartphone] If I actually saved it on the phone it would be useful to actually learn from, because before I went to do my next [clinical evaluation exercise], I could look at my last [one] and go okay, several times doctors have said that I should say this.” [40, p. 928]
**The implementation of mLearning in clinical contexts**		
	Institutional infrastructure and resources	“Loss of carrier signal or connection was a recurring event. … One lecturer described their experience, ‘this week I had a problem with 3G connection, so missed a day using [my tablet] while sorting that out’.” [Educators] [47, p. 4]
		“Several schools talked about the importance of all the sites having Wi-Fi. … [one reported that a] ‘commonly cited reason for our clerkship students to not use them was if they were at a site where the Wi-Fi was unreliable or unavailable’.” [Educators] (30, p. 1154]
	mLearning training and technical support	“[Training could be improved] If the [training workshop] hour was tailored to the tool [mobile device]… interviewing each other did not work… we just talked.” [45, p. 116]
		“…have some base level training…for everybody…specifically on knowing how to turn it on and manipulate it, how it should be used and how it benefits medical education, how the faculty or school expect it to be used. …you need drop-in sessions, extra assistance or individual assistance for people struggling with the technology…” [Educator] [45, p. 116]
	mLearning needs course planning and institutional leadership	“Focus on the areas where you really feel like the [tablet] is an appropriate tool for the thing you want to do, but do not try to wedge [it] into areas where it may or may not be the best thing to use… there are things you can do and things you cannot do at each step along the way.” [Educator] [30, p. 1154]
		“the participants categorised the teachers as being either *old school*, a term they frequently assigned to the older generation, or *new school*, a term afforded to more youthful practitioners—the former being described as *paper-dependent* and being offended when interviewees used their devices in front of them. Many of the *old school* doctors did not appear to understand the reliance that the younger student generation have on their mobile devices as learning tools.” [56, p. 5]
		“It’s things like that [teacher advocacy] which encourage you, maybe I will bring it with me tomorrow and take it on the ward round with me.” [12, p. 7]
		“…it is easy to see the value of some technologies where it works very well and it is very easy to get over-enthusiastic about it and then not realize that people might not be ready to actually use that technology for whatever reasons…” [Educators] [45, p. 116]

^a^mLearning: mobile learning.

**Figure 3:**
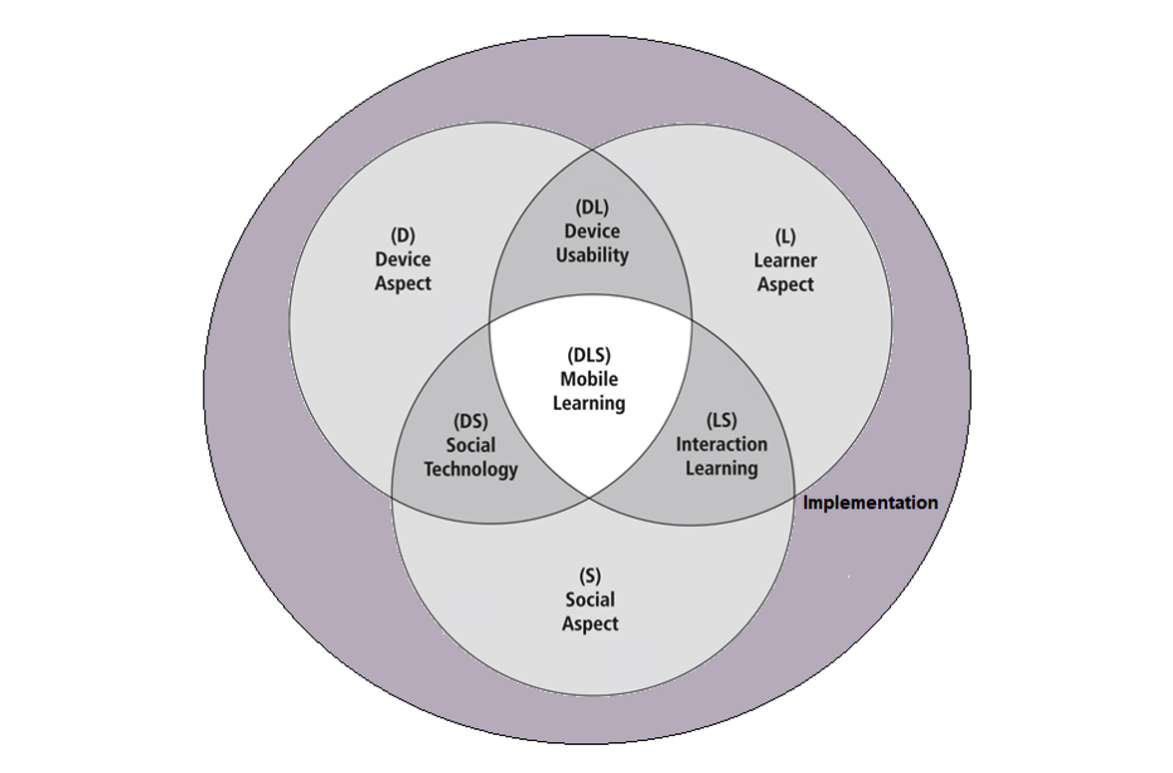
Framework for the Rational Analysis of Mobile Education (FRAME) model adapted for this study.

The FRAME model was adapted to account for these differing findings. Social technology was altered to account for the impact of mobile devices on social interaction, rather than to describe how it enabled connection between multiple interfacing entities. We also added an additional circle that contained the model’s overlapping 3 circles (see the theme *Implementation of mLearning in clinical learning contexts* in Figure 3). Otherwise, the synthesis produced themes that could be grouped under the aspects of learning represented in Koole’s original model, and we used subthemes under each aspect to help illustrate attributes that relate to the specifics of doctors’ and nurses’ learning.

#### Device Usability

Participants referred to the physical, technical, and functional characteristics of mobile devices in relation to an individual’s learning, which involved access, manipulation, and storage of information. Subthemes explored possible positive and unintended consequences of devices being mobile, views on the sufficiency of device functionality, and ideas about the individualistic nature of device use. Enthusiasm for a mobile device focused on efficiency yet was accompanied by an awareness of the need for caution, in terms of a risk of loss of device or contamination in certain settings. Reports of problems attributed to hardware and software were seen in a range of studies, with some participants noting that screens were too small for reading documents [37,40,61] or that they lost information owing to system crashes [12,27,38]. Learners reported wanting devices that suited their own specific needs, describing device use as either *a way of life* (p. 111 [65]) or as a *part of my life now* (p. 1401 [53]).

#### Social Technology

This theme encompassed participants’ perspectives on social responses to mobile devices and many studies were conducted in clinical contexts. Within these settings, students were expected to combine their learning with practice, which resulted in the device influencing social interactions with a number of actors, including their supervisors, patients, and peers. Mobile devices seemed to hold the symbolic value of being a form of technology for recreational use rather than for learning, owing to multiple functions enabling information retrieval alongside highly social activities, such as sending and viewing messages.

Mobile devices, thus, affected students’ relationships with patients and their professional identity. For example, although mobile devices were seen as potentially strengthening communication between clinicians and patients, concerns were raised about possible interference with activities at the bedside. There were reports of feeling *rude* [37,56,58] or *awkward* [31,58]. Although some feared being viewed as unprofessional by either patients or colleagues, others linked device use with perceptions of increased competence. Although these social norms did result in some students being reluctant to openly use the device, others developed strategies for negotiating device use including asking for permission, explaining device use, and jointly using devices with patients.

#### Interaction Learning

Studies highlighted how mobile use enabled learning processes contingent on students’ interaction with their academic institution, peers, and practice. Students used these multiple forms of interaction to learn cooperatively with their peers, organize competing demands of clinical practice and study, and situate their learning within clinical contexts. These forms of mLearning encompassed individual device use for the purposes of information retrieval and organization to device-enabled group work.

Regarding device-enabled group work, online study groups were described as enabling case discussions, and participants commented upon the pros and cons of structured, cooperative peer assessment approaches. It also enabled students to remotely contact supervisors while working in clinical settings. Meanwhile, students and staff described using mobile devices to help them organize their learning, for example, to access information on learning activities when in a clinical setting. As such, students emphasized the value of access to immediately relevant or difficult-to-access clinical cases or using devices to prepare immediately before encounters.

#### Mobile Learning Processes

Some participants reflected upon mLearning as a whole process. Subthemes here represent views on how educational roles could be changing and the process of adapting one’s learning to the mobile device. In terms of the former theme, both students and tutors described how they were participating on more equal terms. Enthusiasm, however, was far from universal, and positive comparisons were made with more traditional forms of learning. Frustration and impatience were expressed about the process of learning how to use a device. Participants described a reliance on others, in particular peers and friends, and although familiarity reportedly improved over time, the need for support and repeat training was emphasized. Uncertainty was voiced over the trustworthiness or reliability of information being distributed through mLearning apps or websites.

#### The Implementation of Mobile Learning in Clinical Contexts

Study participants reported challenges with mLearning that had little to do with interactions between students, devices and their contents, patients, and tutors. Here, what was implicated were insufficient institutional structures and resources, a lack of device-focused training and support, and limited planning and leadership of mLearning programs. For example, the importance—and yet variability—of network connectivity was emphasized by both tutors and students, and concerns were raised over program provision of ill-suited devices.

The use of mLearning strategies did not always appear to have been planned with course content or pedagogy in mind, or with consideration of the attributes required by teaching staff. Students reported they were offered little guidance on how to integrate mobile devices into their learning activities as well as a lack of device knowledge among clinical instructors. Experiential or ongoing training and local technical support were particularly valued; participants reported forgetting functions covered during orientation, and support had been experienced by some as fragmented. Reports were made across numerous studies of disapproval for device use among supervising staff in clinical settings and of students, as a result, being hesitant to use a device openly. A range of proposals were made across the studies, including initiatives to improve staff awareness about the value of portable devices and the development of codes of conduct.

## Discussion

### Principal Findings

To our knowledge, this is the first systematic review synthesizing qualitative research findings about health professionals’ experiences of mLearning. The review identified a total of 47 studies that varied in the types of health professionals involved, their stage of learning, and the mLearning strategies considered. Qualitative data in the majority of studies had been sought so as to pilot mLearning approaches or examine nonspecific use of mobile devices for learning. In many studies, qualitative findings were slim and provided little explanatory detail but across this body of work, it is possible to identify recurring themes about experiences and some explanatory narratives from both learners and educators.

Our synthesis of findings from these studies illustrates some of the potentials of mLearning but also some of the challenging realities for students, doctors, and nurses who are learning in contexts where mobile devices have either formally been introduced or tend to be common. Early commentators on mLearning envisaged methods of delivery that would be *highly suited* to the *just enough, just in time, and just for me* demands of twenty-first century learners [69]. Students in the studies we reviewed did indeed value devices for the possibility of lessening cognitive loads and helping to make good use of time. They also described device use in terms of individualized needs and preferences. However, although both learners and educators described the potential value of devices for accessing, organizing, and enhancing learning, limitations in hardware were reported across the full time period covered by our included studies. Researchers in other spheres of education have also emphasized the need for devices to be fit for purpose [70-72]. Future mLearning strategies for medical and nursing education should, therefore, be developed with an awareness of device affordances for the learning activities required.

This synthesis identifies additional social and institutional factors that seem key for understanding how mLearning for health professionals might be implemented to the best effect. In particular, throughout much of their training, medical and nursing students need to combine learning with professional caring responsibilities. The social aspects of learning that are already complex within more formal education settings become considerably multilayered when students are, for example, at the bedside or in an operating theatre. On top of interactions with information, fellow learners, and formal educators come interactions with a variety of other health professionals and with patients. Learning can happen through peripheral participation in clinical activities, observation, role modeling, and reflective activities, as well as through work with lecturers, supervisors, and other students, and with text books and other information sources. mLearning needs to fit into this mix of interactions but instead our synthesis contains accounts of reluctance, told by both students and educators, toward the use of mobile devices in the clinical workplace because of existing, often implicit, rules for practice. Although negotiation was said sometimes to enable device use for learning, participants in more than one study identified a need for procedural guidance on device use, echoing calls from education more broadly [73,74].

We found Koole’s conceptualization of mLearning, involving a combination of learner, device, and social aspects, to be helpful when organizing findings. However, the themes of social technology and mLearning processes in our synthesis diverged from that of Koole’s conceptualization. With regard to social technology, Koole’s [17] model emphasizes how *mobile devices enable communication and collaboration among multiple individuals and systems* (p. 34), whereas findings from studies within this synthesis instead identify impacts of mLearning on interactions with patients and the management of professional identity.

The FRAME model [17] represents the mLearning processes as an integration of the device, learner, and social aspects that provides for *enhanced collaboration between learners, access to information, and deeper contextualization of learning* (p. 38). Although there were some positive accounts of device use for situated learning and of cooperative learning activities, accounts from studies in our synthesis placed more emphasis on the process of learning how to apply devices for the purposes of learning. Qualitative research into mLearning for health professional education appears still to be in its infancy, with few studies referring to the supported integration of mLearning within a pedagogically informed program of study.

### Limitations

This study provided a comprehensive overview of current qualitative research on mLearning strategies in medical and nursing education. Its strengths include a sensitive search strategy encompassing several bibliographic databases and independent screening by pairs of reviewers, both lowering the likelihood that relevant literature would be overlooked as well as coding and synthesis work done independently and in pairs, aimed at bringing a variety of perspectives to the act of making sense of a heterogeneous set of study findings.

The review is, nevertheless, limited by the qualities of the reviewed studies, especially those employing mixed-methods designs, wherein the quantitative component was given far heavier weighting than qualitative findings. Few studies described in sufficient detail the steps taken by researchers to ensure confidence in the quality of their findings. The majority of studies offered little explanation of methods used to sample participants and collect or analyze data. Moreover, there were many studies in which authors provided little evidence as to how they arrived at their findings. These studies offered few quotes from participants, sometimes making it difficult to decipher whether results were guided by the perspectives of respondents.

Another methodological limitation was that nearly all of the studies explored learning within clinical settings. Owing to this, much of the mLearning described would be classified as informal learning, that is, learning which results from incidental day-to-day activities. Our synthesis, therefore, contains little detail sourced from experiences of programs set up to encourage mLearning in university settings.

Finally, few studies explicitly referred to educators’ learning theories or described course structures in any detail, which meant study findings could not be explored in terms of different objectives for students’ learning. Efforts should be made in future qualitative studies to clearly define the educational purposes of the mLearning programs concerned to make findings more applicable to given learning circumstances.

### Comparison With Earlier Work

Findings about a lack of device training, technical support, and other forms of institutional support led to one of the biggest modifications to the FRAME model seen in our synthesis, which was the development of an additional aspect—implementation in a clinical context. This theme highlighted that even when mobile devices had been introduced for the purpose of evaluation, this appeared to have been done with insufficient consideration of course content or needs at the institutional level, including both sufficient Wi-Fi coverage and the alignment and capacity of teaching staff to use mLearning. Insights might be gained through the study of device maintenance services on campus [45] and the implementation of mLearning strategies with learning outcomes as well as a wider curriculum in mind [30,45,47]. Studies of change management around learning technology in higher education outside the field of health might also be relevant here, as they have explored the potential for initiatives, such as staff as champions, and strategic contextual analyses [75-77].

This review also starts to identify gaps in the literature where additional studies might throw light on a more complete range of mLearning practices within medical and nursing education. For example, study authors made no mention of discussion by study participants of ethical concerns over patient privacy and data security. Educational experts, however, raise concerns vis-à-vis use of mLearning strategies in other settings, arguing that these interventions can compromise students’ confidentiality as private data can be potentially disseminated to unintended audiences [74,78,79]. In terms of medical and nursing education, there is the added concern that the welfare of patients might be compromised. The need identified above for guidance for health professionals’ device use, consequently, will require a strong ethical component.

### Conclusions

The findings of our review have underlined that there is still much to be understood about what is involved in mLearning for medical and nursing education. Our review has indicated that mLearning can potentially play a substantial role as students are already likely to be using mobile devices for a number of differing purposes associated with their learning, ranging from communication with supervisors to organization of tasks. The multipurpose nature of mobile devices means that students can personalize these tools toward their learning needs, which entails a process of learning within itself.

As with any complex tool used for educational purposes, mobile devices should be appropriately incorporated into the structures of academic and medical institutions and steps need to be taken to ensure that learners fully comprehend the functions of each mobile device or app used for learning. These 2 considerations can only be addressed by paying close attention to the process of implementing mLearning strategies in medical and nursing scholarship and the building of an educational infrastructure that enables use of mLearning techniques.

## Appendix

Multimedia Appendix 1 Protocol for the review.

Multimedia Appendix 2 Search strategy.

Multimedia Appendix 3 Details of included studies.

Multimedia Appendix 4 Quality appraisal of studies included in the synthesis.

Multimedia Appendix 5 Full synthesis narrative with linked citations.

## Abbreviations

FRAME: Framework for the Rational Analysis of Mobile Education
mLearning: mobile learning
PDA: Personal Digital Assistant
