# Supplementary material for: Influences on the Implementation of Mobile Learning for Medical and Nursing Education: Qualitative Systematic Review by the Digital Health Education Collaboration
Source: J Med Internet Res. 2019 Feb 28;21(2):e12895. doi: 10.2196/12895 (PMC6416537; doi:10.2196/12895)
Supplement: Multimedia Appendix 2 [file jmir_v21i2e12895_app2.docx]

**Multimedia Appendix 2. Search Strategy**

As is described in the main report, searches were run in two phases.

An initial phase (Part A below), was run across bibliographic database platforms to identify studies that were potentially of any form of eLearning by any health professional, regardless of study design.

A second phase (Part B below), was run within EPPI-Reveiwer (the software used by the review team). This aimed to identify a sub-set of studies: those of mLearning by nursing or medical students or professionals that used a qualitative approach.

**Part A**

**1 MEDLINE (Ovid) Search Strategy**

1. exp education, professional/ not education, veterinary/

2. Education, Predental/

3. Education, Premedical/

4. exp Students, Health Occupations/

5. ((medic* or premedic* or dent* or laborator* or predent* or midwi?e* or nurs* or nutrition* or orthop* or podiat* or pharmac* or psycholog* or psychiatr* or health or healthcare or occupational therap* or physiotherap* or physical therap* or clinical or surg* or radiolog* or obstetric* or gyn?ecolog* or orthodont* or An?esthesi* or Dermatolog* or Oncolog* or Rheumatolog* or Neurolog* or Patholog* or P?ediatric* or Cardiolog* or Urolog*) adj3 (student* or graduate* or undergraduate* or staff or personnel or practitioner* or clerk* or fellow* or internship* or residen* or educat* or train* or novice* or tutor*)).tw,kf.

6. or/1-5

7. Computer-Assisted Instruction/

8. exp Internet/

9. Computer Simulation/

10. Patient Simulation/

11. software/

12. Mobile Applications/

13. User-Computer Interface/

14. Video Games/

15. Web Browser/

16. Education, Distance/

17. Computers/

18. exp Microcomputers/

19. exp Cell Phones/

20. Games, Experimental/

21. exp Models, Anatomic/

22. Audiovisual Aids/

23. Educational Technology/

24. Electronic Mail/

25. exp Telemedicine/

26. Telenursing/

27. Telecommunications/

28. Webcasts/

29. exp Videoconferencing/

30. ((computer* or digital* or hybrid or blended or mixed mode or distance or remote* or electronic or mobile or online* or interactiv* or multimedia or internet or web* or virtual* or game* or gaming or Videogame* or Videogaming) adj3 (classroom* or course* or educat* or instruct* or learn* or lecture* or simulat* or train* or teach* or tutor* or platform*)).tw,kf.

31. (Simulat* adj3 (course* or educat* or instruct* or learn* or train* or teach* or platform* or high-fidelity)).tw,kf.

32. e-learn*.tw,kf.

33. elearn*.tw,kf.

34. m-learn*.tw,kf.

35. mlearn*.tw,kf.

36. smartphone*.tw,kf.

37. smart-phone*.tw,kf.

38. ((mobile or cell) adj2 phone*).tw,kf.

39. iphone*.tw,kf.

40. android*.tw,kf.

41. ipad*.tw,kf.

42. Personal digital assistant*.tw,kf.

43. handheld computer*.tw,kf.

44. Mobile App?.tw,kf.

45. Mobile Application?.tw,kf.

46. webcast*.tw,kf.

47. webinar*.tw,kf.

48. flipped classroom*.tw,kf.

49. Serious game*.tw,kf.

50. Serious gaming.tw,kf.

51. Patient Simulat*.tw,kf.

52. Virtual patient*.tw,kf.

53. ((educat* or instruct* or learn* or simulat* or train* or teach* or interactiv*) adj2 technolog*).tw,kf.

54. Massive Open Online Course?.tw,kf.

55. Mooc?.tw,kf.

56. (Canvas network or Coursera or Coursesites or edx or Futurelearn or iversity or miriada x or moodle or novoed or openlearning or open2study or plato or spoc or udacity or pingpong).tw,kf.

57. or/7-56

58. 6 and 57

59. Education.fs.

60. Education/

61. Teaching/

62. Learning/

63. exp Inservice Training/

64. Curriculum/

65. educat*.tw,kf.

66. learn*.tw,kf.

67. train*.tw,kf.

68. instruct*.tw,kf.

69. teach*.tw,kf.

70. or/59-69

71. Health Personnel/

72. exp Allied Health Personnel/

73. Anatomists/

74. "Coroners and Medical Examiners"/

75. exp Dental Staff/

76. exp Dentists/

77. Health Educators/

78. Infection Control Practitioners/

79. Medical Laboratory Personnel/

80. exp Medical Staff/

81. exp Nurses/

82. exp Nursing Staff/

83. Personnel, Hospital/

84. Pharmacists/

85. exp Physicians/

86. Physician*.tw,kf.

87. Doctor*.tw,kf.

88. Nurs*.tw,kf.

89. Surg*.tw,kf.

90. Health Personnel.tw,kf.

91. healthcare professional*.tw,kf.

92. radiolog*.tw,kf.

93. dentist*.tw,kf.

94. Pharmacist*.tw,kf.

95. Hospital Administrator*.tw,kf.

96. Podiatr*.tw,kf.

97. Psycholog*.tw,kf.

98. Psychiatr*.tw,kf.

99. An?esthesi*.tw,kf.

100. Clinician*.tw,kf.

101. Dermatolog*.tw,kf.

102. General practioner*.tw,kf.

103. Cardiolog*.tw,kf.

104. Oncolog*.tw,kf.

105. Rheumatolog*.tw,kf.

106. Neurolog*.tw,kf.

107. Patholog*.tw,kf.

108. P?ediatric*.tw,kf.

109. Physiotherap*.tw,kf.

110. Physical therap*.tw,kf.

111. Occupational therap*.tw,kf.

112. dieti?ian*.tw,kf.

113. Dietetic*.tw,kf.

114. midwi?e*.tw,kf.

115. nutrition*.tw,kf.

116. orthopti*.tw,kf.

117. obstetric*.tw,kf.

118. gyn?ecolog*.tw,kf.

119. orthodont*.tw,kf.

120. Urolog*.tw,kf.

121. or/71-120

122. Health Occupations/

123. exp Allied Health Occupations/

124. Biomedical Engineering/

125. Chiropractic/

126. exp Dentistry/

127. exp Evidence-Based Practice/

128. exp Medicine/

129. exp Nursing/

130. Dietetics/

131. Optometry/

132. Orthoptics/

133. exp Pharmacology/

134. exp Pharmacy/

135. Podiatry/

136. Psychology, Medical/

137. Serology/

138. Specialization/

139. exp Surgical Procedures, Operative/

140. exp Radiography/

141. or/122-140

142. 121 or 141

143. 57 and 70 and 142

144. Psychomotor Performance/

145. motor skills/

146. ((psychomotor or procedural or technical) adj3 skill*).tw,kf.

147. (psychomotor adj3 performance).tw,kf.

148. or/144-147

149. 6 and 148

150. 58 or 143 or 149

151. limit 150 to yr="1990 -Current"

**2 Embase (Elsevier) search strategy**

#71.153 #71.149 OR #71.151 OR #71.152 AND [1990-2015]/py

#71.152 #71.6 AND #71.148

#71.151 #71.96 AND #71.111 AND #71.150

#71.150 #71.127 OR #71.143

#71.149 #71.6 AND #71.96

#71.148 #71.144 OR #71.145 OR #71.146 OR #71.147

#71.147 'motor performance'/de

#71.146 'psychomotor development'/de

#71.145 'psychomotor activity'/de

#71.144 'psychomotor performance'/de

#71.143 #71.128 OR #71.129 OR #71.130 OR #71.131 OR #71.132 OR #71.133 OR #71.134 OR #71.135 OR #71.136 OR #71.137 OR #71.138 OR #71.139 OR #71.140 OR #71.141 OR #71.142

#71.142 'radiography'/exp

#71.141 'surgery'/exp

#71.140 'pharmaceutics'/de

#71.139 'optometry'/de

#71.138 'nursing'/exp

#71.137 'evidence based nursing'/de

#71.136 'evidence based emergency medicine'/de

#71.135 'evidence based medicine'/de

#71.134 'evidence based dentistry'/de

#71.133 'evidence based practice'/de

#71.132 'biomedical engineering'/de

#71.131 'biomedicine'/exp NOT ('veterinary medicine'/de OR 'telemedicine'/de OR 'visible human project'/de)

#71.130 'paramedical profession'/de

#71.129 'nursing as a profession'/de

#71.128 'medical profession'/de

#71.127 #71.112 OR #71.113 OR #71.114 OR #71.115 OR #71.116 OR #71.117 OR #71.118 OR #71.119 OR #71.120 OR #71.121 OR #71.122 OR #71.123 OR #71.124 OR #71.125 OR #71.126

#71.126 'health educator'/de

#71.125 'paramedical personnel'/exp

#71.124 'nursing home personnel'/de

#71.123 'mental health care personnel'/de

#71.122 'resident'/de

#71.121 'psychotherapist'/de

#71.120 'physician assistant'/de

#71.119 'physician'/exp

#71.118 'medical staff'/de

#71.117 'medical specialist'/de

#71.116 'medical expert'/exp

#71.115 'coroner'/de

#71.114 'medical personnel'/de

#71.113 'hospital personnel'/exp

#71.112 'health care personnel'/de

#71.111 #71.97 OR #71.98 OR #71.99 OR #71.100 OR #71.101 OR #71.102 OR #71.103 OR #71.104 OR #71.105 OR #71.106 OR #71.107 OR #71.108 OR #71.109 OR #71.110

#71.110 'learning'/de

#71.109 'teaching'/de

#71.108 'postgraduate education'/de

#71.107 'postdoctoral education'/de

#71.106 'masters education'/de

#71.105 'interdisciplinary education'/de

#71.104 'in service training'/de

#71.103 'education program'/de

#71.102 'doctoral education'/de

#71.101 'curriculum development'/de

#71.100 'curriculum'/de

#71.99 'continuing education'/de

#71.98 'computerized adaptive testing'/de

#71.97 'education'/de

#71.96 #71.7 OR #71.8 OR #71.9 OR #71.10 OR #71.11 OR #71.12 OR #71.13 OR #71.14 OR #71.15 OR #71.16 OR #71.17 OR #71.18 OR #71.19 OR #71.20 OR #71.21 OR #71.22 OR #71.23 OR #71.24 OR #71.25 OR #71.26 OR #71.27 OR #71.28 OR #71.29 OR #71.30 OR #71.31 OR #71.32 OR #71.33 OR #71.34 OR #71.35 OR #71.36 OR #71.37 OR #71.38 OR #71.39 OR #71.40 OR #71.41 OR #71.42 OR #71.43 OR #71.44 OR #71.45 OR #71.46 OR #71.47 OR #71.48 OR #71.49 OR #71.50 OR #71.51 OR #71.52 OR #71.53 OR #71.54 OR #71.55 OR #71.56 OR #71.57 OR #71.58 OR #71.59 OR #71.60 OR #71.61 OR #71.62 OR #71.63 OR #71.64 OR #71.65 OR #71.66 OR #71.67 OR #71.68 OR #71.69 OR #71.70 OR #71.71 OR #71.72 OR #71.73 OR #71.74 OR #71.75 OR #71.76 OR #71.77 OR #71.78 OR #71.79 OR #71.80 OR #71.81 OR #71.82 OR #71.83 OR #71.84 OR #71.85 OR #71.86 OR #71.87 OR #71.88 OR #71.89 OR #71.90 OR #71.91 OR #71.92 OR #71.93 OR #71.94 OR #71.95

#71.95 'canvas network':ab,ti OR coursera:ab,ti OR coursesites:ab,ti OR edx:ab,ti OR futurelearn:ab,ti OR iversity:ab,ti OR 'miriada x':ab,ti OR moodle:ab,ti OR novoed:ab,ti OR openlearning:ab,ti OR open2study:ab,ti OR plato:ab,ti OR spoc:ab,ti OR udacity:ab,ti OR pingpong:ab,ti

#71.94 mooc:ab,ti OR moocs:ab,ti

#71.93 ('massive open online' NEXT/1 course*):ab,ti

#71.92 ((educat* OR instruct* OR learn* OR simulat* OR train* OR teach* OR interactiv*) NEAR/2 technolog*):ab,ti

#71.91 (virtual NEXT/1 patient*):ab,ti

#71.90 (patient NEXT/1 simulat*):ab,ti

#71.89 (serious NEXT/1 gaming):ab,ti

#71.88 (serious NEXT/1 game*):ab,ti

#71.87 (flipped NEXT/1 classroom*):ab,ti

#71.86 webinar*:ab,ti

#71.85 webcast*:ab,ti

#71.84 (mobile NEXT/1 (application OR applications)):ab,ti

#71.83 (mobile NEXT/1 (app OR apps)):ab,ti

#71.82 (handheld NEXT/1 computer*):ab,ti

#71.81 ('personal digital' NEXT/1 assistant*):ab,ti

#71.80 ipad*:ab,ti

#71.79 android*:ab,ti

#71.78 iphone*:ab,ti

#71.77 ((mobile OR cell) NEAR/2 phone*):ab,ti

#71.76 (smart NEXT/1 phone*):ab,ti

#71.75 smartphone*:ab,ti

#71.74 mlearn*:ab,ti

#71.73 (m NEXT/1 learn*):ab,ti

#71.72 elearn*:ab,ti

#71.71 (e NEXT/1 learn*):ab,ti

#71.70 (simulat* NEAR/3 (course* OR educat* OR instruct* OR learn* OR train* OR teach* OR platform* OR 'high fidelity')):ab,ti

#71.69 (videogaming NEAR/3 (classroom* OR course* OR educat* OR instruct* OR learn* OR lecture* OR simulat* OR train* OR teach* OR tutor* OR platform*)):ab,ti

#71.68 (videogame* NEAR/3 (classroom* OR course* OR educat* OR instruct* OR learn* OR lecture* OR simulat* OR train* OR teach* OR tutor* OR platform*)):ab,ti

#71.67 (gaming NEAR/3 (classroom* OR course* OR educat* OR instruct* OR learn* OR lecture* OR simulat* OR train* OR teach* OR tutor* OR platform*)):ab,ti

#71.66 ('game-based' NEAR/3 (classroom* OR course* OR educat* OR instruct* OR learn* OR lecture* OR simulat* OR train* OR teach* OR tutor* OR platform*)):ab,ti

#71.65 (game* NEAR/3 (classroom* OR course* OR educat* OR instruct* OR learn* OR lecture* OR simulat* OR train* OR teach* OR tutor* OR platform*)):ab,ti

#71.64 ('virtual-reality' NEAR/3 (classroom* OR course* OR educat* OR instruct* OR learn* OR lecture* OR simulat* OR train* OR teach* OR tutor* OR platform*)):ab,ti

#71.63 (virtual* NEAR/3 (classroom* OR course* OR educat* OR instruct* OR learn* OR lecture* OR simulat* OR train* OR teach* OR tutor* OR platform*)):ab,ti

#71.62 ('web-based' NEAR/3 (classroom* OR course* OR educat* OR instruct* OR learn* OR lecture* OR simulat* OR train* OR teach* OR tutor* OR platform*)):ab,ti

#71.61 (web* NEAR/3 (classroom* OR course* OR educat* OR instruct* OR learn* OR lecture* OR simulat* OR train* OR teach* OR tutor* OR platform*)):ab,ti

#71.60 ('internet-based' NEAR/3 (classroom* OR course* OR educat* OR instruct* OR learn* OR lecture* OR simulat* OR train* OR teach* OR tutor* OR platform*)):ab,ti

#71.59 (internet* NEAR/3 (classroom* OR course* OR educat* OR instruct* OR learn* OR lecture* OR simulat* OR train* OR teach* OR tutor* OR platform*)):ab,ti

#71.58 (multimedia NEAR/3 (classroom* OR course* OR educat* OR instruct* OR learn* OR lecture* OR simulat* OR train* OR teach* OR tutor* OR platform*)):ab,ti

#71.57 (interactiv* NEAR/3 (classroom* OR course* OR educat* OR instruct* OR learn* OR lecture* OR simulat* OR train* OR teach* OR tutor* OR platform*)):ab,ti

#71.56 ('online-based' NEAR/3 (classroom* OR course* OR educat* OR instruct* OR learn* OR lecture* OR simulat* OR train* OR teach* OR tutor* OR platform*)):ab,ti

#71.55 (online* NEAR/3 (classroom* OR course* OR educat* OR instruct* OR learn* OR lecture* OR simulat* OR train* OR teach* OR tutor* OR platform*)):ab,ti

#71.54 (mobile NEAR/3 (classroom* OR course* OR educat* OR instruct* OR learn* OR lecture* OR simulat* OR train* OR teach* OR tutor* OR platform*)):ab,ti

#71.53 (electronic NEAR/3 (classroom* OR course* OR educat* OR instruct* OR learn* OR lecture* OR simulat* OR train* OR teach* OR tutor* OR platform*)):ab,ti

#71.52 (remote* NEAR/3 (classroom* OR course* OR educat* OR instruct* OR learn* OR lecture* OR simulat* OR train* OR teach* OR tutor* OR platform*)):ab,ti

#71.51 ('distance-based' NEAR/3 (classroom* OR course* OR educat* OR instruct* OR learn* OR lecture* OR simulat* OR train* OR teach* OR tutor* OR platform*)):ab,ti

#71.50 (distance NEAR/3 (classroom* OR course* OR educat* OR instruct* OR learn* OR lecture* OR simulat* OR train* OR teach* OR tutor* OR platform*)):ab,ti

#71.49 ('mixed mode' NEAR/3 (classroom* OR course* OR educat* OR instruct* OR learn* OR lecture* OR simulat* OR train* OR teach* OR tutor* OR platform*)):ab,ti

#71.48 (blended NEAR/3 (classroom* OR course* OR educat* OR instruct* OR learn* OR lecture* OR simulat* OR train* OR teach* OR tutor* OR platform*)):ab,ti

#71.47 (hybrid NEAR/3 (classroom* OR course* OR educat* OR instruct* OR learn* OR lecture* OR simulat* OR train* OR teach* OR tutor* OR platform*)):ab,ti

#71.46 (digital* NEAR/3 (classroom* OR course* OR educat* OR instruct* OR learn* OR lecture* OR simulat* OR train* OR teach* OR tutor* OR platform*)):ab,ti

#71.45 ('computer-based' NEAR/3 (classroom* OR course* OR educat* OR instruct* OR learn* OR lecture* OR simulat* OR train* OR teach* OR tutor* OR platform*)):ab,ti

#71.44 (computer* NEAR/3 (classroom* OR course* OR educat* OR instruct* OR learn* OR lecture* OR simulat* OR train* OR teach* OR tutor* OR platform*)):ab,ti

#71.43 'vignette'/de

#71.42 'simulator'/de

#71.41 'simulation'/de

#71.40 'radiotherapy simulator'/de

#71.39 'disease simulation'/de

#71.38 'audiovisual aid'/de

#71.37 'visible human project'/de

#71.36 'educational technology'/de

#71.35 'audiovisual equipment'/de

#71.34 'text messaging'/de

#71.33 'personal digital assistant'/de

#71.32 'microcomputer'/de

#71.31 'computer'/de

#71.30 'videoconferencing'/de

#71.29 'telehealth'/exp

#71.28 'webcast'/de

#71.27 'teleconference'/de

#71.26 'telecommunication'/de

#71.25 'social media'/de

#71.24 'mobile phone'/de

#71.23 'e-mail'/de

#71.22 'virtual reality'/de

#71.21 'internet'/de

#71.20 'computer simulation'/de

#71.19 'computer program'/de

#71.18 'web browser'/de

#71.17 'radiotherapy software'/de

#71.16 'orthopedic software'/de

#71.15 'mobile application'/de

#71.14 'imaging software'/de

#71.13 'data analysis software'/de

#71.12 'communication software'/de

#71.11 'anesthesiology software'/de

#71.10 'computer model'/de

#71.9 'virtual reality modeling language'/de

#71.8 'computer interface'/de

#71.7 'internet'/de

#71.6 #71.1 OR #71.2 OR #71.3 OR #71.4 OR #71.5

#71.5 'nursing student'/exp

#71.4 'medical student'/exp

#71.3 'paramedical student'/exp

#71.2 'paramedical education'/exp

#71.1 'medical education'/exp

**3 Cochrane (Wiley) search strategy**

#1 ((medic* or premedic* or dent* or laborator* or predent* or midwi*e* or nurs* or nutrition* or orthop* or podiat* or pharmac* or psycholog* or psychiatr* or health or healthcare or (occupational next/1 therap*) or physiotherap* or (physical next/1 therap*) or clinical or surg* or radiolog* or obstetric* or gyn*ecolog* or orthodont* or An*esthesi* or Dermatolog* or Oncolog* or Rheumatolog* or Neurolog* or Patholog* or P*ediatric* or Cardiolog* or Urolog*) near/3 (student* or graduate* or undergraduate* or staff or personnel or practitioner* or clerk* or fellow* or internship* or residen* or educat* or train* or novice* or tutor*)):ti,ab

#2 ((computer* or digital* or hybrid or blended or "mixed mode" or distance or remote* or electronic or mobile or online* or interactiv* or multimedia or internet or web* or virtual* or game* or gaming or Videogame* or Videogaming) near/3 (classroom* or course* or educat* or instruct* or learn* or lecture* or simulat* or train* or teach* or tutor* or platform*)):ti,ab

#3 (Simulat* near/3 (course* or educat* or instruct* or learn* or train* or teach* or platform* or high-fidelity)):ti,ab

#4 e-learn*:ti,ab

#5 elearn*:ti,ab

#6 m-learn*:ti,ab

#7 mlearn*:ti,ab

#8 smartphone*:ti,ab

#9 smart-phone*:ti,ab

#10 ((mobile or cell) near/2 phone*):ti,ab

#11 iphone*:ti,ab

#12 android*:ti,ab

#13 ipad*:ti,ab

#14 ("Personal digital" next/1 assistant*):ti,ab

#15 (handheld next/1 computer*):ti,ab

#16 (Mobile next/1 App):ti,ab

#17 (Mobile next/1 Apps):ti,ab

#18 (Mobile next/1 Application):ti,ab

#19 (Mobile next/1 Applications):ti,ab

#20 webcast*:ti,ab

#21 webinar*:ti,ab

#22 (flipped next/1 classroom*):ti,ab

#23 (Serious next/1 game*):ti,ab

#24 (Serious next/1 gaming):ti,ab

#25 (Patient next/1 Simulat*):ti,ab

#26 (Virtual next/1 patient*):ti,ab

#27 ((educat* or instruct* or learn* or simulat* or train* or teach* or interactiv*) near/2 technolog*):ti,ab

#28 ("Massive Open Online" next/1 Course*):ti,ab

#29 Mooc:ti,ab

#30 Moocs:ti,ab

#31 ("Canvas network" or Coursera or Coursesites or edx or Futurelearn or iversity or "miriada x" or moodle or novoed or openlearning or open2study or plato or spoc or udacity or pingpong):ti,ab

#32 {or #2-#31}

#33 #1 and #32

#34 educat*:ti,ab

#35 learn*:ti,ab

#36 train*:ti,ab

#37 instruct*:ti,ab

#38 teach*:ti,ab

#39 {or #34-#38}

#40 Physician*:ti,ab

#41 Doctor*:ti,ab

#42 Nurs*:ti,ab

#43 Surg*:ti,ab

#44 "Health Personnel":ti,ab

#45 (healthcare next/1 professional*):ti,ab

#46 radiolog*:ti,ab

#47 dentist*:ti,ab

#48 Pharmacist*:ti,ab

#49 (Hospital next/1 Administrator*):ti,ab

#50 Podiatr*:ti,ab

#51 Psycholog*:ti,ab

#52 Psychiatr*:ti,ab

#53 An*esthesi*:ti,ab

#54 Clinician*:ti,ab

#55 Dermatolog*:ti,ab

#56 (General next/1 practioner*):ti,ab

#57 Cardiolog*:ti,ab

#58 Oncolog*:ti,ab

#59 Rheumatolog*:ti,ab

#60 Neurolog*:ti,ab

#61 Patholog*:ti,ab

#62 P*ediatric*:ti,ab

#63 Physiotherap*:ti,ab

#64 (Physical next/1 therap*):ti,ab

#65 (Occupational next/1 therap*):ti,ab

#66 dieti*ian*:ti,ab

#67 Dietetic*:ti,ab

#68 midwi*e*:ti,ab

#69 nutrition*:ti,ab

#70 orthopti*:ti,ab

#71 obstetric*:ti,ab

#72 gyn*ecolog*:ti,ab

#73 orthodont*:ti,ab

#74 Urolog*:ti,ab

#75 {or #40-#74}

#76 #32 and #39 and #75

#77 ((psychomotor or procedural or technical) near/3 skill*):ti,ab

#78 (psychomotor near/3 performance):ti,ab

#79 #77 or #78

#80 #1 and #79

#81 #33 or #76 or #80 Publication Year from 1990 to 2015

**4 PsycInfo (Ovid) search strategy**

1. exp graduate education/

2. nursing education/

3. exp Psychology Education/

4. exp Clinical Methods Training/

5. medical students/

6. nursing students/

7. dental students/

8. therapist trainees/

9. ((medic* or premedic* or dent* or laborator* or predent* or midwi?e* or nurs* or nutrition* or orthop* or podiat* or pharmac* or psycholog* or psychiatr* or health or healthcare or occupational therap* or physiotherap* or physical therap* or clinical or surg* or radiolog* or obstetric* or gyn?ecolog* or orthodont* or An?esthesi* or Dermatolog* or Oncolog* or Rheumatolog* or Neurolog* or Patholog* or P?ediatric* or Cardiolog* or Urolog*) adj3 (student* or graduate* or undergraduate* or staff or personnel or practitioner* or clerk* or fellow* or internship* or residen* or educat* or train* or novice* or tutor*)).tw,id.

10. or/1-9

11. exp Computer Assisted Instruction/

12. internet/

13. exp social media/

14. computer mediated communication/

15. exp Computer Simulation/

16. simulation/

17. computer software/

18. computer applications/

19. computer games/

20. simulation games/

21. websites/

22. distance education/

23. learning management systems/

24. computers/

25. instructional media/

26. teaching machines/

27. microcomputers/

28. cellular phones/

29. exp audiovisual instruction/

30. educational audiovisual aids/

31. telemedicine/

32. telecommunications media/

33. teleconferencing/

34. ((computer* or digital* or hybrid or blended or mixed mode or distance or remote* or electronic or mobile or online* or interactiv* or multimedia or internet or web* or virtual* or game* or gaming or Videogame* or Videogaming) adj3 (classroom* or course* or educat* or instruct* or learn* or lecture* or simulat* or train* or teach* or tutor* or platform*)).tw,id.

35. (Simulat* adj3 (course* or educat* or instruct* or learn* or train* or teach* or platform* or high-fidelity)).tw,id.

36. e-learn*.tw,id.

37. elearn*.tw,id.

38. m-learn*.tw,id.

39. mlearn*.tw,id.

40. smartphone*.tw,id.

41. smart-phone*.tw,id.

42. ((mobile or cell) adj2 phone*).tw,id.

43. iphone*.tw,id.

44. android*.tw,id.

45. ipad*.tw,id.

46. Personal digital assistant*.tw,id.

47. handheld computer*.tw,id.

48. Mobile App?.tw,id.

49. Mobile Application?.tw,id.

50. webcast*.tw,id.

51. webinar*.tw,id.

52. flipped classroom*.tw,id.

53. Serious game*.tw,id.

54. Serious gaming.tw,id.

55. Patient Simulat*.tw,id.

56. Virtual patient*.tw,id.

57. ((educat* or instruct* or learn* or simulat* or train* or teach*) adj3 technolog*).tw,id.

58. Massive Open Online Course?.tw,id.

59. Mooc?.tw,id.

60. (Canvas network or Coursera or Coursesites or edx or Futurelearn or iversity or miriada x or moodle or novoed or openlearning or open2study or plato or spoc or udacity or pingpong).tw,id.

61. or/11-60

62. 10 and 61

63. education/

64. exp continuing education/

65. higher education/

66. postgraduate training/

67. teaching/

68. teaching methods/

69. learning/

70. curriculum/

71. curriculum development/

72. educat*.tw,id.

73. learn*.tw,id.

74. train*.tw,id.

75. instruct*.tw,id.

76. teach*.tw,id.

77. or/63-76

78. exp Health Personnel/

79. clinicians/

80. exp psychologists/

81. therapists/

82. Physician*.tw,id.

83. Doctor*.tw,id.

84. Nurs*.tw,id.

85. Surg*.tw,id.

86. Health Personnel.tw,id.

87. healthcare professional*.tw,id.

88. radiolog*.tw,id.

89. dentist*.tw,id.

90. Pharmacist*.tw,id.

91. Hospital Administrator*.tw,id.

92. Podiatr*.tw,id.

93. Psycholog*.tw,id.

94. Psychiatr*.tw,id.

95. An?esthesi*.tw,id.

96. Clinician*.tw,id.

97. Dermatolog*.tw,id.

98. General practioner*.tw,id.

99. Cardiolog*.tw,id.

100. Oncolog*.tw,id.

101. Rheumatolog*.tw,id.

102. Neurolog*.tw,id.

103. Patholog*.tw,id.

104. P?ediatric*.tw,id.

105. Physiotherap*.tw,id.

106. Physical therap*.tw,id.

107. Occupational therap*.tw,id.

108. dieti?ian*.tw,id.

109. Dietetic*.tw,id.

110. midwi?e*.tw,id.

111. nutrition*.tw,id.

112. orthopti*.tw,id.

113. obstetric*.tw,id.

114. gyn?ecolog*.tw,id.

115. orthodont*.tw,id.

116. Urolog*.tw,id.

117. or/78-116

118. exp paramedical sciences/

119. evidence based practice/

120. exp medical sciences/

121. exp psychology/

122. exp neuroimaging/

123. or/118-122

124. 117 or 123

125. 61 and 77 and 124

126. perceptual motor processes/

127. perceptual motor coordination/

128. exp perceptual motor learning/

129. motor skills/

130. ((psychomotor or procedural or technical) adj3 skill*).tw,id.

131. (psychomotor adj3 performance).tw,id.

132. or/126-131

133. 10 and 132

134. 62 or 125 or 133

135. limit 134 to yr="1990 -Current"

**5 ERIC (Ovid) search strategy**

1. exp medical education/ not veterinary education/

2. allied health occupations education/

3. medical students/

4. premedical students/

5. nursing students/

6. ((medic* or premedic* or dent* or laborator* or predent* or midwi?e* or nurs* or nutrition* or orthop* or podiat* or pharmac* or psycholog* or psychiatr* or health or healthcare or occupational therap* or physiotherap* or physical therap* or clinical or surg* or radiolog* or obstetric* or gyn?ecolog* or orthodont* or An?esthesi* or Dermatolog* or Oncolog* or Rheumatolog* or Neurolog* or Patholog* or P?ediatric* or Cardiolog* or Urolog*) adj3 (student* or graduate* or undergraduate* or staff or personnel or practitioner* or clerk* or fellow* or internship* or residen* or educat* or train* or novice* or tutor*)).tw.

7. or/1-6

8. exp Computer uses in education/

9. exp internet/

10. Computer Simulation/

11. computer software/

12. computer interfaces/

13. video games/

14. computer games/

15. Web Browsers/

16. distance education/

17. exp computers/

18. exp handheld devices/

19. exp audiovisual aids/

20. educational technology/

21. electronic mail/

22. telecommunications/

23. exp teleconferencing/

24. electronic learning/

25. Computer Mediated Communication/

26. Blended Learning/

27. ((computer* or digital* or hybrid or blended or mixed mode or distance or remote* or electronic or mobile or online* or interactiv* or multimedia or internet or web* or virtual* or game* or gaming or Videogame* or Videogaming) adj3 (classroom* or course* or educat* or instruct* or learn* or lecture* or simulat* or train* or teach* or tutor* or platform*)).tw.

28. (Simulat* adj3 (course* or educat* or instruct* or learn* or train* or teach* or platform* or high-fidelity)).tw.

29. e-learn*.tw.

30. elearn*.tw.

31. m-learn*.tw.

32. mlearn*.tw.

33. smartphone*.tw.

34. smart-phone*.tw.

35. ((mobile or cell) adj2 phone*).tw.

36. iphone*.tw.

37. android*.tw.

38. Personal digital assistant*.tw.

39. handheld computer*.tw.

40. Mobile App?.tw.

41. Mobile Application?.tw.

42. webcast*.tw.

43. webinar*.tw.

44. flipped classroom*.tw.

45. Serious game*.tw.

46. Serious gaming.tw.

47. Patient Simulat*.tw.

48. Virtual patient*.tw.

49. ((educat* or instruct* or learn* or simulat* or train* or teach* or interactiv*) adj2 technolog*).tw.

50. Massive Open Online Course?.tw.

51. Mooc?.tw.

52. (Canvas network or Coursera or Coursesites or edx or Futurelearn or iversity or miriada x or moodle or novoed or openlearning or open2study or plato or spoc or udacity or pingpong).tw.

53. or/8-52

54. 7 and 53

55. education/

56. exp Higher Education/

57. exp Continuing Education/

58. teaching methods/

59. Learning/

60. inservice education/

61. Staff Development/

62. curriculum/

63. curriculum development/

64. educat*.tw.

65. learn*.tw.

66. train*.tw.

67. instruct*.tw.

68. teach*.tw.

69. or/55-68

70. exp Health Personnel/

71. physician*.tw.

72. doctor*.tw.

73. nurs*.tw.

74. surg*.tw.

75. health personnel.tw.

76. healthcare professional*.tw.

77. radiolog*.tw.

78. dentist*.tw.

79. Pharmacist*.tw.

80. Hospital Administrator*.tw.

81. Podiatr*.tw.

82. Psychiatr*.tw.

83. Psycholog*.tw.

84. An?esthesi*.tw.

85. Clinician*.tw.

86. Dermatolog*.tw.

87. General practioner

88. Cardiolog*.tw.

89. Oncolog*.tw.

90. Rheumatolog*.tw.

91. Neurolog*.tw.

92. Patholog*.tw.

93. P?ediatric*.tw.

94. Physiotherap*.tw.

95. Physical therap*.tw.

96. Occupational therap*.tw.

97. dieti?ian*.tw.

98. Dietetic*.tw.

99. midwi?e*.tw.

100. nutrition*.tw.

101. orthopti*.tw.

102. obstetric*.tw.

103. gyn?ecolog*.tw.

104. orthodont*.tw.

105. Urolog*.tw.

106. or/70-105

107. exp Health Occupations/

108. exp Medicine/ not Veterinary Medicine/

109. optometry/

110. psychology/

111. specialization/

112. Radiograph*.tw.

113. or/107-112

114. 106 or 113

115. 53 and 69 and 114

116. psychomotor skills/

117. Perceptual Motor Coordination/

118. ((psychomotor or procedural or technical) adj3 skill*).tw.

119. (psychomotor adj3 performance).tw.

120. or/116-119

121. 7 and 120

122. 54 or 115 or 121

123. limit 122 to yr="1990 -Current"

**6 Cinahl (Ebsco) search strategy**

| S1 | (MH "Education, Health Sciences+") |
| --- | --- |
| S2 | (MH "Education, Premedical") |
| S3 | (MH "Education, Clinical+") |
| S4 | (MH "Students, Health Occupations+" OR MH "Students, Pre-Nursing") |
| S5 | TI((medic* or premedic* or dent* or laborator* or predent* or midwife or midwives or nurs* or nutrition* or orthop* or podiat* or pharmac* or psycholog* or psychiatr* or health or healthcare or "occupational therap*" or physiotherap* or "physical therap*" or clinical or surg* or radiolog* or obstetric* or gynecolog* or gynaecolog* or orthodont* or anesthesi*or anaesthesi* or Dermatolog* or Oncolog* or Rheumatolog* or Neurolog* or Patholog* or pediatric* or paediatric* or Cardiolog* or Urolog*) N3 (student* or graduate* or undergraduate* or staff or personnel or practitioner* or clerk* or fellow* or internship* or residen* or educat* or train* or novice* or tutor*)) OR AB ((medic* or premedic* or dent* or laborator* or predent* or midwife or midwives or nurs* or nutrition* or orthop* or podiat* or pharmac* or psycholog* or psychiatr* or health or healthcare or "occupational therap*" or physiotherap* or "physical therap*" or clinical or surg* or radiolog* or obstetric* or gynecolog* or gynaecolog* or orthodont* or anesthesi* or anaesthesi* or Dermatolog* or Oncolog* or Rheumatolog* or Neurolog* or Patholog* or pediatric* or paediatric* or Cardiolog* or Urolog*) N3 (student* or graduate* or undergraduate* or staff or personnel or practitioner* or clerk* or fellow* or internship* or residen* or educat* or train* or novice* or tutor*)) |
| S6 | S1 OR S2 OR S3 OR S4 OR S5 |

| S7 | | (MH "Computer Assisted Instruction") |  |
| --- | --- | --- | --- |
| S8 | | (MH "Internet" OR MH "Social Media" OR MH "World Wide Web Applications") |  |
| S9 | | (MH Virtual Reality) |  |
| S10 | | (MH "Software") |  |
| S11 | | (MH "User-Computer Interface") |  |
| S12 | | (MH "Video Games") |  |
| S13 | | (MH "Web Browsers") |  |
| S14 | | (MH "Education, Non-Traditional") |  |
| S15 | | (MH "Videoconferencing") |  |
| S16 | | (MH "Microcomputers+") |  |
| S17 | | (MH "Educational Technology") |  |
| S18 | | (MH "Simulations+") |  |
| S19 | | (MH "Models, Anatomic+") |  |
| S20 | | (MH "Audiovisuals") |  |
| S21 | | (MH "Electronic Mail") |  |
| S22 | | (MH "Telehealth+") |  |
| S23 | | (MH "Telecommunications") |  |
| S24 | | TI((computer* or digital* or hybrid or blended or "mixed mode" or distance or remote* or electronic or mobile or online* or interactiv* or multimedia or internet or web* or virtual* or game* or gaming or videogame* or videogaming) N3 (classroom* or course* or educat* or instruct* or learn* or lecture* or simulat* or train* or teach* or tutor* or platform*)) OR AB ((computer* or digital* or hybrid or blended or "mixed mode" or distance or remote* or electronic or mobile or online* or interactiv* or multimedia or internet or web* or virtual* or game* or gaming or videogame* or videogaming) N3 (classroom* or course* or educat* or instruct* or learn* or lecture* or simulat* or train* or teach* or tutor* or platform*)) |  |
| S25 | | TI(Simulat* N3 (course* or educat* or instruct* or learn* or train* or teach* or platform* or "high-fidelity")) OR AB (Simulat* N3 (course* or educat* or instruct* or learn* or train* or teach* or platform* or "high-fidelity")) |  |
| S26 | | TI e-learn* OR AB e-learn* |  |
| S27 | | TI elearn* OR AB elearn* |  |
| S28 | | TI m-learn* OR AB m-learn* |  |
| S29 | | TI mlearn* OR AB mlearn* |  |
| S30 | | TI smartphone* OR AB smartphone* |  |
| S31 | | TI smart-phone* OR AB smart-phone* |  |
| S32 | | TI ( ((mobile or cell) N2 phone*) ) OR AB ( ((mobile or cell) N2 phone*) ) |  |
| S33 | | TI iphone* OR AB iphone* |  |
| S34 | | TI android* OR AB android* |  |
| S35 | | TI ipad* OR AB ipad* |  |
| S36 | | TI "Personal digital assistant*" OR AB "Personal digital assistant*" |  |
| S37 | | TI "handheld computer*" OR AB "handheld computer*" |  |
| S38 | | TI "Mobile App" OR AB "Mobile App" OR TI "Mobile Apps" OR AB "Mobile Apps" |  |
| S39 | | TI "Mobile Application" OR AB "Mobile Application" OR TI "Mobile Applications" OR AB "Mobile Applications" |  |
| S40 | | TI webcast* OR AB webcast* |  |
| S41 | | TI webinar* OR AB webinar* |  |
| S42 | | TI "flipped classroom*" OR AU "flipped classroom*" |  |
| S43 | | TI "serious game*" OR AB "serious game*" |  |
| S44 | | TI "serious game*" OR AB "serious game*" |  |
| S45 | | TI "serious gaming" OR AB "serious gaming" |  |
| S46 | | TI "patient simulat*" OR AB "patient simulat*" |  |
| S47 | | TI "virtual patient*" OR AB "virtual patient*" |  |
| S48 | TI((educat* or instruct* or learn* or simulat* or train* or teach* or interactiv*) N2 technolog*) OR AB ((educat* or instruct* or learn* or simulat* or train* or teach* or interactiv*) N2 technolog*) | | |
| S49 | TI "Massive Open Online Course*" OR AB "Massive Open Online Course*" | | |
| S50 | TI Mooc OR AB Mooc OR TI Moocs OR AB Moocs | | |
| S51 | TI (("Canvas network" or Coursera or Coursesites or edx or Futurelearn or iversity or "miriada x" or moodle or novoed or openlearning or open2study or plato or spoc or udacity or pingpong) ) OR AB (("Canvas network" or Coursera or Coursesites or edx or Futurelearn or iversity or "miriada x" or moodle or novoed or openlearning or open2study or plato or spoc or udacity or pingpong) ) | | |
| S52 | S7 OR S8 OR S9 OR S10 OR S11 OR S12 OR S13 OR S14 OR S15 OR S16 OR S17 OR S18 OR S19 OR S20 OR S21 OR S22 OR S23 OR S24 OR S25 OR S26 OR S27 OR S28 OR S29 OR S30 OR S31 OR S32 OR S33 OR S34 OR S35 OR S36 OR S37 OR S38 OR S39 OR S40 OR S41 OR S42 OR S43 OR S44 OR S45 OR S46 OR S47 OR S48 OR S49 OR S50 OR S51 | | |
| S53 | S6 AND S52 | | |
| S54 | (MW "ed") | | |
| S55 | (MH "Education") | | |
| S56 | (MH "Teaching") | | |
| S57 | (MH "Learning") | | |
| S58 | (MH "Staff Development+" OR MH "Refresher Courses") | | |
| S59 | (MH "Curriculum" OR MH "Curriculum Development" ) | | |
| S60 | TI educat* OR AB educat* | | |
| S61 | TI learn* OR AB learn* | | |
| S62 | TI train* OR AB train* | | |
| S63 | TI instruct* OR AB instruct* | | |
| S64 | TI teach* OR AB teach* | | |
| S65 | S54 OR S55 OR S56 OR S57 OR S58 OR S59 OR S60 OR S61 OR S62 OR S63 OR S64 | | |
| S66 | (MH "Health Personnel") | | |
| S67 | (MH "Allied Health Personnel+") | | |
| S68 | (MH "Alternative Health Personnel+") | | |
| S69 | (MH "Nurses+") | | |
| S70 | (MH "Personnel, Health Facility+") | | |
| S71 | (MH "Pharmacists") | | |
| S72 | (MH "Midwives+") | | |
| S73 | (MH "Physicians+") | | |
| S74 | (MH "Operating Room Personnel+") | | |
| S75 | TI physician* OR AB physician* | | |
| S76 | TI doctor* OR AB doctor* | | |
| S77 | TI nurs* OR AB nurs* | | |
| S78 | TI surg* OR AB surg* | | |
| S79 | TI "health personnel*" OR AB "health personnel*" | | |
| S80 | TI "healthcare professional*" OR AB "healthcare professional*" | | |
| S81 | TI radiolog* OR AB radiolog* | | |
| S82 | TI dentist* OR AB dentist* | | |
| S83 | TI pharmacist* OR AB pharmacist* | | |
| S84 | TI "hospital administrator*" OR AB "hospital administrator*" | | |
| S85 | TI podiatr* OR AB podiatr* | | |
| S86 | TI psycholog* OR AB psycholog* | | |
| S87 | TI psychiatr* OR AB psychiatr* | | |
| S88 | TI anesthesi* OR AB anesthesi* OR TI anaesthesi* OR AB anaesthesi* | | |
| S89 | TI clinician* OR AB clinician* | | |
| S90 | TI dermatolog* OR dermatolog* | | |
| S91 | TI "General practioner*" OR AB "General practioner*" | | |
| S92 | TI cardiolog* OR AB cardiolog* | | |
| S93 | TI oncolog* OR AB oncolog* | | |
| S94 | TI rheumatolog* OR AB rheumatolog* | | |
| S95 | TI neurolog* OR AB neurolog* | | |
| S96 | TI patholog* OR AB patholog* | | |
| S97 | TI pediatric* OR AB pediatric* OR TI paediatric* OR AB paediatric* | | |
| S98 | TI physiotherap* OR AB physiotherap* | | |
| S99 | TI "physical therap*" OR AB "physical therap*" | | |
| S100 | TI "occupational therap*" OR AB "occupational therap*" | | |
| S101 | TI dietician* OR AB dietician* OR TI dietitian* AB dietitian* | | |
| S102 | TI dietetic* OR AB dietetic* | | |
| S103 | TI midwife OR AB midwife OR TI midwives OR AB midwives | | |
| S104 | TI nutrition* OR AB nutrition* | | |
| S105 | TI orthopti* OR AB orthopti* | | |
| S106 | TI obstetric* OR AB obstetric* | | |
| S107 | TI gynecolog* OR AB gynecolog* OR TI gynaecolog* OR AB gynaecolog* | | |
| S108 | TI orthodont* OR AB orthodont* | | |
| S109 | TI urolog* OR AB urolog* | | |
| S110 | S66 OR S67 OR S68 OR S69 OR S70 OR S71 OR S72 OR S73 OR S74 OR S75 OR S76 OR S77 OR S78 OR S79 OR S80 OR S81 OR S82 OR S83 OR S84 OR S85 OR S86 OR S87 OR S88 OR S89 OR S90 OR S91 OR S92 OR S93 OR S94 OR S95 OR S96 OR S97 OR S98 OR S99 OR S100 OR S101 OR S102 OR S103 OR S104 OR S105 OR S106 OR S107 OR S108 OR S109 | | |
| S111 | (MH "Health Occupations") | | |
| S112 | (MH "Allied Health Professions") | | |
| S113 | (MH "Biomedical Engineering") | | |
| S114 | (MH "Chiropractic") | | |
| S115 | (MH "Dentistry+") | | |
| S116 | (MH "Professional Practice, Evidence-Based+") | | |
| S117 | (MH "Medicine+") | | |
| S118 | (MH "Nursing Care+") | | |
| S119 | (MH "Audiology") OR (MH "Dental Hygiene") OR (MH "Dietetics") OR (MH "Emergency Medical Technicians") OR (MH "Medical Assisting") OR (MH "Physician Assistants") OR (MH "Public Health Nutrition") OR (MH "Speech-Language Pathology") OR (MM "Technology, Radiologic") | | |
| S120 | (MH "Optometry") OR (MH "Podiatry") OR (MH "Serology") OR (MH "Specialization") | | |
| S121 | (MH "Pharmacy and Pharmacology+") | | |
| S122 | (MH "Radiography+") | | |
| S123 | (MH "Surgery, Operative+") | | |
| S124 | (MH "Midwifery+") | | |
| S125 | S111 OR S112 OR S113 OR S114 OR S115 OR S116 OR S117 OR S118 OR S119 OR S120 OR S121 OR S122 OR S123 OR S124 | | |
| S126 | S110 OR S125 | | |
| S127 | S52 AND S65 AND S126 | | |
| S128 | (MH "Psychomotor Performance") OR (MH "Motor Skills") | | |
| S129 | (MH "Psychomotor Performance") OR (MH "Motor Skills") | | |
| S130 | TI ( ((psychomotor or procedural or technical) N3 skill*) ) OR AB ( ((psychomotor or procedural or technical) N3 skill*) ) | | |
| S131 | TI (psychomotor N3 performance) OR AB (psychomotor N3 performance) | | |
| S132 | S128 OR S129 OR S130 OR S131 | | |
| S133 | S6 AND S132 | | |
| S134 | S53 OR S127 OR S133 | | |
| S135 | S53 OR S127 OR S133  Limiters - Published Date: 19900101-20151231 | | |

**7 Web of Science Core Collection (Thomson Reuters) search strategy**

**#1** TS=(((medic* or premedic* or dent* or laborator* or predent* or midwi*e* or nurs* or nutrition* or orthop* or podiat* or pharmac* or psycholog* or psychiatr* or health or healthcare or “occupational therap*” or physiotherap* or “physical therap*” or clinical or surg* or radiolog* or obstetric* or gyn*ecolog* or orthodont* or An*esthesi* or Dermatolog* or Oncolog* or Rheumatolog* or Neurolog* or Patholog* or P*ediatric* or Cardiolog* or Urolog*) NEAR/3 (student* or graduate* or undergraduate* or staff or personnel or practitioner* or clerk* or fellow* or internship* or residen* or educat* or train* or novice* or tutor*)))
**#2** TS= (((computer* or digital* or hybrid or blended or “mixed mode” or distance or remote* or electronic or mobile or online* or interactiv* or multimedia or internet or web* or virtual* or game* or gaming or Videogame* or Videogaming) NEAR/3 (classroom* or course* or educat* or instruct* or learn* or lecture* or simulat* or train* or teach* or tutor* or platform*)))

**#3** TS= (((Simulat*) NEAR/3 (course* or educat* or instruct* or learn* or train* or teach* or platform* or “high-fidelity”))
**#4** TS= (((educat* or instruct* or learn* or simulat* or train* or teach* or interactiv*) NEAR/2 (technolog*)))
**#5** TS=(“Canvas network” or Coursera or Coursesites or edx or Futurelearn or iversity or "miriada x" or moodle or novoed or openlearning or open2study or plato or spoc or udacity or pingpong or "Massive Open Online Course*" or Mooc or Moocs or e-learn* or elearn* or m-learn* or mlearn* or smartphone* or smart-phone* or iphone* or android* or ipad* or “personal digital assistant*” or “handheld computer*” or “mobile app” or “mobile apps” or “mobile application” or “mobile applications” or webcast* or webinar* or “flipped classroom*” or “serious game*” or “serious gaming” or “patient simulat*” or “virtual patient*” or ((mobile or cell) adj2 phone*))

**#6** #5 OR #4 OR #3 OR #2
**#7** #6 AND #1

**#8** TS=((educat* or teach* or learn* or train* instruct*))

**#9** TS=((physician* or doctor* or nurs* or surg* or “health personnel” or “healthcare professional*” or radiolog* or dentist* or pharmacist* or “hospital administrator*” or podiatr* or psycholog* or psychiatr* or an*esthesi* or clinician* or dermatolog* or “general practioner*” or cardiolog* or oncolog* or rheumatolog* or neurolog* or patholog* or p*ediatric* or physiotherap* or “physical therap*” or “occupational therap*” dieti*ian* or dietetic* or midwi*e* or nutrition* or orthopti* or obstetric* or gyn*ecolog* or orthodont* or urolog*))

**#10** #9 AND #8 AND #6

**#11** TS=(((psychomotor or procedural or technical) NEAR/3 (skill*))) OR TS=(((psychomotor) NEAR/3 (performance))

**#12** #11 AND #1

**#13** #12 OR #10 OR #7

#14 #12 OR #10 OR #7
Refined by: **PUBLICATION YEARS:** (2013 OR 2001 OR 2012 OR 1998 OR 2014 OR 1999 OR 2011 OR 1997 OR 2010 OR 1996 OR 2009 OR 1995 OR 2008 OR 1994 OR 2007 OR 1993 OR 2006 OR 2015 OR 2005 OR 1992 OR 2004 OR 1991 OR 2003 OR 1990 OR 2002 OR 2000)

**8 International Clinical Trials Platform (ICTRP) WHO**

Source Link: <http://apps.who.int/trialsearch/AdvSearch.aspx>

Data sets from [data providers](http://www.who.int/ictrp/search/data_providers/en/index.html) are updated every Wednesday evening according to the following schedule:
Every week:

- Australian New Zealand Clinical Trials Registry, last data file imported on **24 October 2016**
- Chinese Clinical Trial Registry, last data file imported on **24 October 2016**
- ClinicalTrials.gov, last data file imported on **24 October 2016**
- EU Clinical Trials Register (EU-CTR), last data file imported on **24 October 2016**
- ISRCTN, last data file imported on **24 October 2016**
- The Netherlands National Trial Register, last data file imported on **25 October 2016**

Every 4 weeks:

- Brazilian Clinical Trials Registry (ReBec), last data file imported on **11 October 2016**
- Clinical Trials Registry - India, last data file imported on **10 October 2016**
- Clinical Research Information Service - Republic of Korea, last data file imported on **10 October 2016**
- Cuban Public Registry of Clinical Trials, last data file imported on **11 October 2016**
- German Clinical Trials Register, last data file imported on **10 October 2016**
- Iranian Registry of Clinical Trials, last data file imported on **10 October 2016**
- Japan Primary Registries Network, last data file imported on **10 October 2016**
- Pan African Clinical Trial Registry, last data file imported on **10 October 2016**
- Sri Lanka Clinical Trials Registry, last data file imported on **10 October 2016**
- Thai Clinical Trials Register (TCTR), last data file imported on **18 October 2016**
- ****New**** Peruvian Clinical Trials Registry (REPEC), last data file imported on **11 October 2016**

**SEARCH 1**

Using the title field in the advanced search, searching ALL registered trials

e-learning OR elearning OR m-learning OR mlearning OR "Canvas network" OR Coursera OR Coursesites OR edx OR Futurelearn OR iversity OR "miriada x" OR moodle OR novoed OR openlearning OR open2study OR plato OR spoc OR udacity OR pingpong OR "Massive Open Online Course*" OR Mooc OR Moocs OR smartphone* OR smart-phone* OR iphone* OR android* OR ipad* OR "personal digital assistant*" OR "handheld computer*" OR "mobile app" OR "mobile apps" OR "mobile application" OR "mobile applications" OR webcast* OR webinar* OR "flipped classroom*" OR "serious game*" OR "serious gaming" OR "patient simulat*" OR "virtual patient*" OR "psychomotor performance"

**SEARCH 2**

Using the title field in the advanced search, searching ALL registered trials

(comput* OR digital* OR hybrid OR blended OR "mixed mode" OR distance OR remote* OR electronic OR mobile OR online* OR interactiv* OR multimedia OR internet OR web* OR virtual OR game* OR gaming OR videogame* OR videogaming OR simulat* OR virtual OR technolog*)

AND

(classroom* OR course* OR educat* OR instruct* OR learn* OR lecture* OR simulat* OR train* OR teach* OR tutor* OR platform* OR "high-fidelity")

**Part B**

Searches run in EPPI-Reviewer 4

123 122 AND 112 AND 62

122 120 OR 119 OR 118 OR 117 OR 116 OR 115 OR 114 OR 121 OR 87

121 "clinician*" (in Title and Abstract)

120 "physician*" (in Title and Abstract)

119 "clerkship" (in Title and Abstract)

118 ""nurs* student*"" (in Title and Abstract)

117 ""med* student*"" (in Title and Abstract)

116 "resident*" (in Title and Abstract)

115 "nurse*" (in Title and Abstract)

114 "doctor*" (in Title and Abstract)

113 112 AND 87 AND 62

112 70 OR 111 OR 110 OR 109 OR 108 OR 107 OR 106 OR 105 OR 104 OR 103 OR 102 OR 101 OR 100 OR 99 OR 98 OR 97 OR 96 OR 95 OR 94 OR 93 OR 92 OR 91 OR 90 OR 89 OR 72 OR 71 OR 69 OR 68 OR 67 OR 66 OR 65 OR 64 OR 63

111 "cell phone" (in Title and Abstract)

110 "wireless device" (in Title and Abstract)

109 "mobile electronic device" (in Title and Abstract)

108 "tablet personal computer" (in Title and Abstract)

107 "tablet PC" (in Title and Abstract)

106 "videogame console" (in Title and Abstract)

105 "video-game console" (in Title and Abstract)

104 "ultraportable computer" (in Title and Abstract)

103 "ultra-portable computer" (in Title and Abstract)

102 "hand-held computer" (in Title and Abstract)

101 "handheld computer" (in Title and Abstract)

100 "hand-held device" (in Title and Abstract)

99 "handheld device" (in Title and Abstract)

98 "portable media player" (in Title and Abstract)

97 "EDA" (in Title and Abstract)

96 "enterprise digital assistant" (in Title and Abstract)

95 "pocket computer" (in Title and Abstract)

94 "smart-phone" (in Title and Abstract)

93 "smartphone" (in Title and Abstract)

92 "PDA" (in Title and Abstract)

91 "personal digital assistant" (in Title and Abstract)

90 "mobile phone" (in Title and Abstract)

89 "mobile" (in Title and Abstract)

88 74 AND 87

87 75 OR 76 OR 77 OR 78 OR 79 OR 80 OR 81 OR 82 OR 83 OR 84 OR 85 OR 86

86 "postgraduate nursing education" (in Title and Abstract)

85 "post-registration nursing education" (in Title and Abstract)

84 "post-professional nursing education" (in Title and Abstract)

83 "undergraduate nursing education" (in Title and Abstract)

82 "pre-registration nursing education" (in Title and Abstract)

81 "pre-professional nursing education" (in Title and Abstract)

80 "postgraduate medical education" (in Title and Abstract)

79 "post-registration medical education" (in Title and Abstract)

78 "post-professional medical education" (in Title and Abstract)

77 "undergraduate medical education" (in Title and Abstract)

76 "pre-registration medical education" (in Title and Abstract)

75 "pre-professional medical education" (in Title and Abstract)

74 62 AND 73

73 72 OR 71 OR 70 OR 69 OR 68 OR 67 OR 66 OR 65 OR 64 OR 63

72 "mobile tutoring" (in Title and Abstract)

71 "mobile coaching" (in Title and Abstract)

70 "mobile teaching" (in Title and Abstract)

69 "mobile schooling" (in Title and Abstract)

68 "mobile training" (in Title and Abstract)

67 "mobile instruction" (in Title and Abstract)

66 "mobile education" (in Title and Abstract)

65 "m-learning" (in Title and Abstract)

64 "mlearning" (in Title and Abstract)

63 "mobile learning" (in Title and Abstract)

62 61 OR 60 OR 59 OR 58 OR 57 OR 56 OR 55 OR 54 OR 53 OR 52 OR 51 OR 50 OR 49 OR 48 OR 47 OR 46 OR 45 OR 44 OR 43 OR 42 OR 41 OR 40 OR 39 OR 38 OR 37 OR 36 OR 35 OR 34 OR 33 OR 32 OR 31 OR 30 OR 29 OR 28 OR 27 OR 26 OR 25 OR 24 OR 23 OR 22 OR 21 OR 20 OR 19 OR 18 OR 17 OR 16 OR 15 OR 14 OR 13 OR 12 OR 11 OR 10 OR 9 OR 8 OR 7 OR 6 OR 5 OR 4 OR 3 OR 2 OR 1

61 "openended" (in Title and Abstract)

60 "open-ended" (in Title and Abstract)

59 "opinions" (in Title and Abstract)

58 "opinion" (in Title and Abstract)

57 "beliefs" (in Title and Abstract)

56 "belief" (in Title and Abstract)

55 "attitude" (in Title and Abstract)

54 "attitudes" (in Title and Abstract)

53 "feeling" (in Title and Abstract)

52 "feelings" (in Title and Abstract)

51 "discourses" (in Title and Abstract)

50 "discourse" (in Title and Abstract)

49 "stories" (in Title and Abstract)

48 "story" (in Title and Abstract)

47 "case study" (in Title and Abstract)

46 "case studies" (in Title and Abstract)

45 "field research" (in Title and Abstract)

44 "field studies" (in Title and Abstract)

43 "field study" (in Title and Abstract)

42 "narrated" (in Title and Abstract)

41 "narratives" (in Title and Abstract)

40 "narrative" (in Title and Abstract)

39 "narrative research" (in Title and Abstract)

38 "thematic analysis" (in Title and Abstract)

37 "content analysis" (in Title and Abstract)

36 "action research" (in Title and Abstract)

35 "grounded research" (in Title and Abstract)

34 "grounded studies" (in Title and Abstract)

33 "grounded study" (in Title and Abstract)

32 "grounded theory" (in Title and Abstract)

31 "ethnographical" (in Title and Abstract)

30 "ethnography" (in Title and Abstract)

29 "phenomenological" (in Title and Abstract)

28 "phenomenology" (in Title and Abstract)

27 "focus groups" (in Title and Abstract)

26 "focus group" (in Title and Abstract)

25 "interviewer" (in Title and Abstract)

24 "interviewed" (in Title and Abstract)

23 "interview" (in Title and Abstract)

22 "interviews" (in Title and Abstract)

21 "experiences" (in Title and Abstract)

20 "experience" (in Title and Abstract)

19 "perception" (in Title and Abstract)

18 "perceptions" (in Title and Abstract)

17 "perceived" (in Title and Abstract)

16 "perceives" (in Title and Abstract)

15 "perceive" (in Title and Abstract)

14 "perspectives" (in Title and Abstract)

13 "perspective" (in Title and Abstract)

12 "view" (in Title and Abstract)

11 "views" (in Title and Abstract)

10 "experimental" (in Title and Abstract)

9 "experiment" (in Title and Abstract)

8 "causal research" (in Title and Abstract)

7 "correlational research" (in Title and Abstract)

6 "survey" (in Title and Abstract)

5 "multi method" (in Title and Abstract)

4 "multi-method" (in Title and Abstract)

3 "mixed method" (in Title and Abstract)

2 "mixed-method" (in Title and Abstract)

1 "qualitative" (in Title and Abstract)
