# Supplementary material for: Influences on the Implementation of Mobile Learning for Medical and Nursing Education: Qualitative Systematic Review by the Digital Health Education Collaboration
Source: J Med Internet Res. 2019 Feb 28;21(2):e12895. doi: 10.2196/12895 (PMC6416537; doi:10.2196/12895)
Supplement: Multimedia Appendix 3 [file jmir_v21i2e12895_app3.docx]

Multimedia Appendix 3 - Details of included studies (N =47).

| Study, reference number | Type of study and aims | Country, population | Type of study and learning | Methods | Overall quality weights |
| --- | --- | --- | --- | --- | --- |
| Akkerman and Filuius, 2011 [25] | Interventional study examining the role of PDAs in supporting work-based learning during internships | The Netherlands, medical students as co-assistants | Personal learning enabled through a number of apps on participants’ PDAs, eg, note-taking software | Mixed methods, including individual interviews | Medium, medium |
| Armstrong et al 2012 [26] | Interventional study evaluating the use of *txt2MEDLINE* as short message service (SMS) query of PubMed/MEDLINE, & SMS text messaging optimized clinical guidelines | Botswana, doctors, nurses, medical students, residents, and other health care workers | Messaging services through SMS text messaging | Mixed methods, including focus groups | Low, Low |
| Avila et al 2016 [27] | Interventional study evaluating an ePortfolio system with mobile capabilities | Germany, medical students | Personal learning enabled through a number of apps managed via the WordPress ePortfolio system | Mixed methods, including focus groups | Low, Low |
| Axelson et al 2007 [28] | Interventional study exploring junior doctors’ experiences of using medical knowledge sources on PDAs | Sweden, junior doctors | Reference repository provided through ProHospital and SafeMed Pocket | Focus groups | High, medium |
| Davies et al 2012 [12] | Interventional study examining how participants engage with mLearning, as well as barriers and facilitators to its use | United Kingdom, medical students | Reference repository provided through a suite of apps | Mixed methods, including focus groups & free-text responses within the survey | High, High |
| Davies et al 2014 [29] | Interventional study exploring efﬁcacy of using mobile technology in facilitating increased levels of interaction and group cohesion within a series of tutorial sessions involving undergraduate nursing students | United Kingdom, nursing students | Multimodal learning enabled through various apps, for example, Whiteboard HD and DropBox | Mixed methods, including free-text comments on the survey | Medium, high |
| Deutsch et al 2016 [30] | Inquiry of experiences of using mobile devices that sought to identify clinical uses and limitations of mobile technology in the clinical curriculum | United States, senior academic and medical staff | No specified type of learning | Individual interviews | Medium, medium |
| Dimond et al 2016 [31] | Interventional study examining how a mobile textbook app could support newly-qualified doctors in providing safe and efficient patient care | United Kingdom, junior doctors | Reference repository provided through Dr Companion software with access to medical texts | Web-based questionnaire with open-ended questions. | Medium, high |
| Doyle et al 2016 [32] | Interventional study exploring how nursing students use mobile technology to support learning | Canada, nursing students | Reference repository providing access to standard nursing guides, Medline journals and various clinical calculators | Web-based questionnaire with open-ended questions. | Low, low |
| Ellaway et al 2014 [33] | Interventional study investigating participants' patterns of mobile usage, barriers, and facilitators to mLearning as well as how this form of learning interacted with the wider educational environment | Canada, medical students | Undirected use of PDA for learning, participants given access to Lexi-Complete | Mixed methods, including focus groups and case study methods | Medium, low |
| Evangelinos, 2014 [34] | Inquiry of experiences of using mobile devices that assessed the applicability of the EU digital competence framework within medical and nursing education | United Kingdom, unspecified health care trainees and academic professionals | No specified type of learning | Mixed methods, including individual interviews | Low, low |
| Fahlman and Holley, 2013 [35] | Inquiry of experiences of using mobile devices that explored use of mobile devices for the purposes of continual learning for registered nurses | Canada, nurses | No specified type of learning | Mixed methods, including individual interview | Medium, medium |
| Fan et al 2016 [36] | Inquiry of experiences of using mobile devices that examined types of Web 2.0 tools and mobile devices being used by potential digiMe users, as well as reasons for adoption | Australia, medical students and educators | No specified type of learning | Mixed methods, including survey with open-ended questions and individual interviews | Medium, low |
| Farrell and Rose, 2008 [37] | Interventional study investigating whether PDAs could enhance students' pharmacological and clinical contextual knowledge and identify issues associated with the use of PDAs | Australia, nursing students | Reference repository, including The Monthly Index of Medical Specialties | Mixed methods, including focus groups | Low, low |
| Garrett and Jackson, 2015 [38] | Interventional study exploring an initial proof-of-concept design in using augmented reality resources to supplement clinical skills lab teaching | Canada, nursing students | Augmented reality technologies | Mixed methods, including focus groups | Medium, high |
| Garrett and Jackson, 2006 [39] | Interventional study evaluating a PDA-based tool to support reﬂective learning in practice in a pilot study | Canada, nursing and medical students | Personal learning enabled through a number of apps, including an ePortfolio tool | Mixed methods, including focus groups | Medium, Medium |
| Green et al 2015 [40] | Interventional study evaluating students’ perceptions regarding MBChB mobile | United Kingdom, medical students | Personal learning enabled through a number of apps, including MBChB mobile program | Mixed methods, including focus groups | Medium, low |
| Hardyman et al 2013 [10] | Interventional study assessing the value and impact of a smartphone library of texts to support workplace learning | United Kingdom, trainee doctors | Reference repository | Mixed methods, including case study | Low, medium |
| Jamu et al 2016 [41] | Interventional study evaluating the effectiveness, acceptability, and feasibility of Quick Response (QR) codes for the purposes of multiprofessional practice-based learning | United Kingdom, student nurses, health care assistants, registered nurses, medical students, and doctors | Reference repository accessed through QR codes | Mixed methods, including individual interview | Medium, low |
| Jang and Kim, 2014 [42] | Interventional study investigating students' experiences of using mLearning to acquire clinical skills | South Korea, medical students | Video tutorials (asynchronous) on objective structured clinical examination | Mixed methods, including individual interview | Low, low |
| Kucuk et al 2016 [43] | Interventional study examining effectiveness of an augmented reality app for assisting students with learning anatomy | Turkey, medical students | Augmented reality provided through smartphone app | Mixed methods, including interviews | Medium, low |
| Kumar et al 2011 [44] | Interventional study exploring experience of learners using mobile devices to support learning | India, learners in post graduate diploma in clinical cardiology program and program-in-charges | SMS text messaging and undirected use of PDA through singular app | Mixed methods, including individual interview | Low, low |
| Luanrattana et al 2012 [45] | Pre-intervention/needs-based study examining feasibility of incorporating PDAs into a PBL-based medical curriculum | Australia, senior medical and academic staff | No specified type of learning. Students used various apps used for PBL-based medical curriculum | Individual interviews | Medium, medium |
| MacKay and Harding, 2009 [46] | Interventional study assessing the value of using SMS text messaging to support nursing students’ workplace learning | New Zealand, nursing students and lecturer | Messaging services provided through eTXT telecommunication technology and short message texting service | Mixed methods, including lecturers’ field notes & students’ responses to teaching evaluation | Low, low |
| Mackay et al 2017 [47] | Interventional study describing the process of intervention and exploring clinical nurse lecturers’ perceptions and experience of the use of mobile smart devices to support student learning | New Zealand, nursing lecturers | Personal learning enabled through a number of apps, for example, reference apps | Reflective journal and focus groups | High, medium |
| Mather and Cummings, 2015 [48] | Inquiry of experiences of using mobile devices that explored current mLearning strategies employed by clinical supervisors to augment learning in tertiary and community-based health care settings in 2 Australian states | Australia, clinical supervisors | No specified type of learning | Focus groups | Low, low |
| Mather and Cummings, 2016 [2] | Inquiry of experiences of using mobile devices that explored nursing students’ perception of the opportunities and barriers related to use of mLearning strategies | Australia, nursing students | No specified type of learning. Students used a variety of apps (eg, medication management resources) | Mixed-methods, including survey with open-ended questions | Low, low |
| Nuss et al 2014 [49] | Interventional study examining medical students’ use of the iPad during their Internal Medicine clerkship | United States of America, medical students | Reference repository provided through a variety of apps and bookmark links to the medical school library, PubMed and MedlinePlus | Mixed methods, including individual interviews | Medium, high |
| O’Connor and Andrews, 2016 [50] | Pre-intervention/needs-based study that examined the co-design process for creating a clinical skills–based smartphone app and identified functions nursing students need in a personalized educational app | Ireland, nursing students | No specified type of learning | Co-design workshops and focus groups | Medium, medium |
| O’Donovan and Maruthappu, 2016 [51] | Pre-intervention/needs-based study that assessed the feasibility and impact of using PDAs to deliver video tutorials and remote online peer-tutoring between 2 countries | Malaysia, medical students | No specified type of learning, students used open-access online video tutorials, pdf materials, and videoconferencing via Skype | Mixed-methods, including survey with open-ended questions and individual interviews | High, medium |
| Pilcher and Bedford, 2011 [52] | Inquiry of experiences of using mobile devices that explored nurses’ technological preferences for learning & examined factors associated with their willingness to use educational technology | United States, nurses | No specified type of learning | Mixed-methods, including individual interviews | High, medium |
| Pimmer et al 2014 [53] | Inquiry of experiences of using mobile devices that examined how nurses use mobile technology to enable learning in marginalized and remote areas | South Africa, nurses, facilitators, and clinical managers | No specified type of learning | Individual interviews | High, high |
| Pimmer et al 2013 [54] | Inquiry of experiences of using mobile devices that explored use and impact of educational technology in medical education | Nepal, medical students, teachers, and faculty | No specified type of learning | Focus groups | High, high |
| Prakash et al 2016 [55] | Interventional study evaluating efficacy of an Android-based App, *AIIMS-WHO CC STPs*, which acts as a point-of-care tool | India, doctors | Multimodal learning provided through a suit of apps, including *AIIMS-WHO CC STPs* and other apps involving algorithmic management of common conditions in sick neonates. | Mixed methods, including focus groups | Low, medium |
| Rashid-Doubell et al 2016 [56] | Inquiry of experiences of using mobile devices that examined senior students' experiences of mLearning in a clinical setting and identified the challenges of using mobile devices in these settings | Bahrain, medical students | No specified type of learning | Individual interviews | Medium, high |
| Rusatira et al 2016 [67] | Pre-intervention/needs-based study that explored user requirements and possible educational resources | Rwanda, doctors | No specified type of learning | Mixed-methods, including individual interviews | Low, medium |
| Sergeeva et al 2016 [57] | Inquiry of informal learning using mobile devices that identified intended and unintended effects of use of mobile devices on health care work practices of nurses. | The Netherlands, operating room nurses | Personal learning enabled through a number of apps | Participant observation and individual interviews | Low, high |
| Strayer et al 2010 [58] | Interventional study examining how a PDA-based smoking cessation counseling tool influences medical student smoking cessation counseling | Australia, medical students | A behavior-prompting app, E-SMOKE-I.T., that was loaded onto students' PDA | Mixed-methods, including focus groups | High, medium |
| Thukral et al 2014 [59] | Interventional study evaluating efficacy of an interactive app as a training tool on newborn care for postgraduate nursing students | India, nursing students | Multimodal learning provided through a training tool on mobile phone or PDA | Mixed methods, including focus groups | High, medium |
| Varcadipane et al 2015 [60] | Interventional study exploring medical residents’ patterns of iPad usage & their opinions on the use of such devices in medical practice | United States, medical residents | Undirected use of PDA for learning through various apps on participants’ iPad | Mixed methods, including focus groups | Medium, low |
| Wang, Wiesemes and Gibbons, 2012 [61] | Interventional study examining whether mobile devices influence doctoral students’ time management or learning | United Kingdom, doctoral research nursing students | Personal learning enabled through a number of apps, a Web-based blackboard learning discussion forum & an online asynchronous conference | Group meeting notes, small online questionnaire, & exit interviews | Medium, medium |
| Wells, 2014 [62] | Interventional study exploring neurology residents’ experiences of participating in a supplementary sleep medicine eLearning module | United States, medical residents | Personal and interactive learning enabled through a number of apps, including Sleep 101 module - an eLearning module with a streaming video lecture series, online discussion board, and authentic sleep medicine resources | Case study methods | High, high |
| Willemse, 2015 [63] | Interventional study exploring undergraduate nursing students’ experiences of using WhatsApp to supplement primary health care education | South Africa, nursing students | Social media group enabled through WhatsApp | Electronic reflections of participants | Low, high |
| Witt et al 2016 [68] | Interventional study analyzing which apps were most useful to undergraduate medical students, determining how devices and the infrastructure could be optimized to encourage their use | Botswana, medical students | Reference repository provided through a variety of apps, including Medscape, Epocrates, Skyscape Medical Resources, & PubMed Mobile. | Mixed methods, including focus groups | Low, high |
| Wu, 2014 [64] | Interventional study examining nursing students’ perception and acceptance of a mobile learning system | Taiwan, nursing students and teaching staff | Multimodal learning enabled through Android apps, such as Google, mountain view audio and video files as annotations | Mixed methods, including individual interviews | Low, low |
| Wyatt et al 2010 [65] | Interventional study exploring how mLearning techniques influence nursing education in multiple locations and discuss the relationship between mLearning and students' learning style | United States, nurses and nursing students | Personal reflection enabled through a number of apps on a Windows Mobile operating system with apps | Mixed-methods, including focus groups | Medium, low |
| Young et al 2010 [66] | Interventional study investigating whether texting provides additional means of support for students during practice placements | United Kingdom, nursing, occupational therapy, and radiography students | Messaging services provided through SMS text messaging | Mixed-methods, including individual interviews | Medium, low |
