# Supplementary material for: Influences on the Implementation of Mobile Learning for Medical and Nursing Education: Qualitative Systematic Review by the Digital Health Education Collaboration
Source: J Med Internet Res. 2019 Feb 28;21(2):e12895. doi: 10.2196/12895 (PMC6416537; doi:10.2196/12895)
Supplement: Multimedia Appendix 4 [file jmir_v21i2e12895_app4.docx]

|  | **Trustworthines of Findings** | | | | **Usefulness of Findings** | | | |
| --- | --- | --- | --- | --- | --- | --- | --- | --- |
| **Study, reference no.** | Sampling | Data collection | Analysis | *Overall weight* | Findings supported by the data | Depth achieved in the findings | Perspectives privileged | *Overall weight* |
| Akkerman and Filuius 2011 [25] | Low | Medium | Medium | *Medium* | High | Medium | Low | *Medium* |
| Armstrong et al. 2012 [26] | Medium | Low | Low | *Low* | Low | Low | Low | *Low* |
| Avila et al. 2016 [27] | Medium | Low | Low | *Low* | Low | Low | Medium | *Low* |
| Axelson et al. 2007 [28] | High | Medium | High | *High* | Low | High | Medium | *Medium* |
| Davies et al. 2012 [12] | Low | High | High | *High* | High | High | Medium | *High* |
| Davies et al. 2014 [29] | Low | Low | High | *Medium* | High | High | Low | *High* |
| Deutsch et al. 2016 [30] | High | Medium | Medium | *Medium* | Medium | Medium | Medium | *Medium* |
| Dimond et al. 2016 [31] | Medium | Medium | High | *Medium* | High | High | Medium | *High* |
| Doyle et al. 2016 [32] | Medium | Low | Low | *Low* | Low | Low | Medium | *Low* |
| Ellaway et al. 2014 [33] | Low | High | High | *Medium* | Low | Medium | Low | *Low* |
| Evangelinos 2014 [34] | Low | Low | Low | *Low* | Low | Low | Medium | *Low* |
| Fahlman and Holley 2013 [35] | High | High | High | *Medium* | Low | Medium | Medium | *Medium* |
| Fan et al. 2016 [36] | Medium | Low | Medium | *Medium* | Low | Low | Medium | *Low* |
| Farrell and Rose 2008 [37] | Low | Medium | Low | *Low* | Medium | Low | Low | *Low* |
| Garrett and Jackson 2006 [39] | Low | Medium | High | *Medium* | High | High | Medium | *High* |
| Garrett and Jackson 2015 [38] | Low | Medium | High | *Medium* | High | Medium | Medium | *Medium* |
| Green et al. 2015 [40] | Medium | Medium | Medium | *Medium* | High | Low | Low | *Low* |
| Hardyman et al. 2013 [10] | Low | Low | Medium | *Low* | High | Medium | Medium | *Medium* |
| Jamu et al. 2016 [41] | Medium | High | Medium | *Medium* | High | Low | Low | *Low* |
| Jang and Kim 2014 [42] | Low | Medium | Low | *Low* | Low | Low | Low | *Low* |
| Kucuk et al. 2016 [43] | Low | High | Low | *Medium* | Medium | Low | Low | *Low* |
| Kumar et al. 2011 [44] | Medium | Low | Low | *Low* | Low | Low | Low | *Low* |
| Luanrattana et al. 2012 [45] | Medium | Medium | High | *Medium* | Medium | High | Low | *Medium* |
| MacKay and Harding 2009 [46] | Low | Low | Low | *Low* | Low | Low | Low | *Low* |
| Mackay et al. 2017 [47] | Low | High | High | *High* | Medium | Medium | Medium | *Medium* |
| Mather and Cummings 2015 [48] | Low | Low | Low | *Low* | High | Low | Low | *Low* |
| Mather and Cummings 2016 [2] | Low | Low | Medium | *Low* | High | Low | Low | *Low* |
| Nuss et al. 2014 [49] | Low | Medium | High | *Medium* | High | High | Low | *High* |
| O’Connor and Andrews 2016 [50] | Low | Medium | High | *Medium* | Low | Medium | Medium | *Medium* |
| O’Donovan and Maruthappu 2016 [51] | High | High | Low | *High* | Medium | Medium | Low | *Medium* |
| Pilcher and Bedford 2011 [52] | High | High | High | *High* | Medium | High | Low | *Medium* |
| Pimmer et al. 2013 [54] | Medium | High | High | *High* | High | High | Medium | *High* |
| Pimmer et al. 2014 [53] | Medium | High | High | *High* | High | High | Medium | *High* |
| Prakash et al. 2016 [55] | Medium | Low | Low | *Low* | Medium | Low | Medium | *Medium* |
| Rashid-Doubell et al. 2016 [56] | Medium | Medium | High | *Medium* | High | High | Medium | *High* |
| Rusatira et al. 2016 [67] | Medium | Low | Medium | *Low* | Low | High | Low | *Medium* |
| Sergeeva et al. 2016 [57] | Low | Low | High | *Low* | High | High | Low | *High* |
| Strayer et al. 2010 [58] | High | Medium | High | *High* | High | Medium | Medium | *Medium* |
| Thukral et al. 2014 [59] | High | High | High | *High* | Low | Medium | High | *Medium* |
| Varcadipane et al. 2015 [60] | Low | Medium | High | *Medium* | Low | Low | Low | *Low* |
| Wang, Wiesemes and Gibbons 2012 [61] | Low | Medium | High | *Medium* | Low | High | Medium | *Medium* |
| Wells 2014 [62] | High | Medium | High | *High* | High | High | High | *High* |
| Willemse 2015 [63] | Low | Low | High | *Low* | High | High | Low | *High* |
| Witt et al. 2016 [68] | Low | Low | High | *Low* | High | High | Medium | *High* |
| Wu 2014 [64] | Low | Low | Low | *Low* | Low | Low | Low | *Low* |
| Wyatt et al. 2010 [65] | Medium | Medium | Medium | *Medium* | Low | Low | Low | *Low* |
| Young et al. 2010 [66] | Low | Medium | High | *Medium* | High | Low | Low | *Low* |

Appendix S3: Quality Appraisal
