# Supplementary material for: Influences on the Implementation of Mobile Learning for Medical and Nursing Education: Qualitative Systematic Review by the Digital Health Education Collaboration
Source: J Med Internet Res. 2019 Feb 28;21(2):e12895. doi: 10.2196/12895 (PMC6416537; doi:10.2196/12895)
Supplement: Multimedia Appendix 5 [file jmir_v21i2e12895_app5.docx]

# **Full synthesis narrative**

# **Device Usability**

This section presents participants’ references to the physical, technical and functional characteristics of mobile devices in relation to an individual’s learning, and to the accessing, manipulation and storage of information in particular. Sub-themes relate to possible positive and unintended consequences of devices being mobile, views on the sufficiency of device functionality, and ideas about the individualistic nature of device-use.

## Portability means efficiency but also vigilance

Enthusiasm for a mobile device often centred around time saving that could lead to improved learning, and was more than once summed up with the phrase ‘efficient and effective’ [2, 36]. Participants emphasised the speed with which reference material could be retrieved for various purposes [28, 35, 56], but also the quick enabling of dialogue with others (face-to-face as well as virtually), and the rapid storage of material in varied forms (e.g. text and images) for later use [54]. In clinical settings students valued being able to reduce their reliance on memory, both of things that needed to be recalled, and of gaps in knowledge that needed to be remedied [40, 49]. As one put it, ‘it’s a lot easier [to] look up things there and then instead of trying to remember to go back and read up on it.’ [40] (p927).

The portable nature of the mobile device was, also, referred to as a source of vigilance by students and their tutors, who discussed the need for care in surgical or post-operation scenarios and possible electromagnetic interference from devices or the risk of contamination [45, 57]. Other potential threats to the device were voiced in terms of loss [12, 40, 59, 68], damage if it was on loan [40] or theft [12, 60], especially as there was a concern that mobile usage itself could “attract thieves” (p.75)[68].

## Fit for purpose hardware, software and data

Reports of problems attributed to hardware and software were seen in a range of studies. Reported criticisms of hardware included screens being too small for reading documents [37, 40, 61], devices being ill suited for note taking [25, 33, 39, 60, 62] and screens having a poor resolution [51]. Size and weight were also emphasised as important for portability [33, 61].

Software was not always judged as sufficiently intuitive [12, 27], or could interfere with an important learning task, such as self-assessment [40]. The ability of software to transfer and synchronize data or files with a desktop computer or between platforms [28, 61] was given importance in more than one study, as were experiences with technical issues. The latter included devices freezing or stopping [12, 27], the loss of information with system crashes [12, 27, 38], difficulties with attachments or scanning [27, 38] and system incompatibility with platforms or applications [45, 65].

## Ownership, personalisation and sense of self

Despite costs, some students preferred to own devices because this provided opportunities to engage with technology in an informal manner [34, 38], or at times that suited them [28]. In one study where devices were provided, learners who already owned a device preferred to continue to use their own [33]. Across several studies participants emphasised the value of being able to personalise the learning content [28, 36, 50, 68] or programme layout [27] within mobile devices to suit their own needs.

The perceived personal significance of mobile devices for learning is intimated by students using phrases to sum-up PDA or smartphone use such as, ‘a way of life’ (p. 111)[65] and ‘it is part of my life now’(p. 1401) [53], as well as others’ concerns about developing over dependency on their device [12, 28, 33, 34, 56, 59]. Linking their device use to their own cognitive capacity, some were concerned about technology failure [12, 59] or that their loss of recall ability would be problematic during exams [33].

# **Social Technology**

This section focuses on participants’ perspectives on social responses to mobile devices. Many studies were conducted in clinical contexts, wherein students were expected to combine their learning with practice, resulting in the device influencing social interactions with a number of actors, including their supervisors, patients and peers. Mobile devices seemed to hold the symbolic value of being a form of technology for recreational use rather than for learning, owing to its multiple functions enabling information retrieval alongside highly social activities, such as sending and viewing messages. Themes within social technology relate to impacts on students’ relationships with patients and their professional identity, as well as how students sought to negotiate device use.

## Devices can impact on care and learning relationships

Although students [33] and tutors [48] noted that use of mobile devices could potentially strengthen communication between clinicians and patients; both these groups expressed a diversity of concerns related to the perceived inhibiting effects of the devices on interactions with patients. Use of mobile devices was seen by trainee doctors and nurses as interfering with activities at the bedside [56, 57], specifically with medical consultations, clinical observations [56], and teamwork [57]. Some voiced reservations in using mobile devices in front of patients [12, 41, 56] as they felt ‘rude’ [37, 56, 58], ‘awkward’ [31, 58], were wary of appearing ‘insincere or disingenuous’ [56], or felt discomfort due to a lack of technological skills [25]. Not being able to maintain good eye contact with patients was reported as causing difficulties with conversation [56, 58]. Consequently, mobile device use presented a challenge to students building relationships between themselves and other actors [31, 56]. In some instances, reluctance for device use remained despite encouragement from senior clinical staff [41, 67].

## Devices raise issues of professionalism and practice boundaries

Some students feared they would be viewed as unprofessional by either patients or colleagues because devices were perceived as being purely for leisure [12, 31, 34, 40, 56]. Some reported concerns that mobile devices were actually being used for non-work-related and recreational purposes during worktime [56, 57]. Others, however, perceived that use of mobile devices could strengthen their own professional identity or that of their institution [33] or saw others as competent if they used devices to retrieve information [35].

In terms of practice boundaries, there was some discussion of how mobile technology may blur the boundaries between clinicians’ personal and professional spaces through the increased availability of work-related information. One student described colleagues as ‘actual prisoners to their phones’ [56].

## Negotiating the social aspect of mobile technology

Students described how it could help if patients were given explanations for device use [31, 40, 48] and this was also described as helpful for managing the responses of clinical supervisors [33]. Some students described how it was possible to actively negotiate digital device use [56, 60, 68], e.g. through asking patients’ permission before using a device [40, 50, 56]. Some described jointly looking up information with patients [31, 48].

# **Interaction Learning**

This section contains findings on how mobile use enabled learning processes contingent on students’ interaction with their academic institution, peers and practice. Through these interactions learners made meaning of experiences within the clinical context, which was, therefore, inescapably social. Sub-themes described how these multiple forms of interaction enabled students to learn cooperatively with their peers, organise competing demands of clinical practice and study to enable learning, and situate their learning within clinical contexts.

## Facilitated interaction and learning

As many of the studies were of devices mainly aimed at supporting information retrieval and storage, students’ accounts often focused upon interactions between themselves and information supplied by their respective institution, or creatable by a device. Valued content included: textbooks, up to date medical literature [28] and clinical guidelines [12, 26, 28, 31, 37, 45, 62, 68]. This type of content enabled students to seek information via mobile devices which was supportive of clinical practice, especially in the absence of more senior advisors [10].

In a few cases, students accessed online study groups developed by tutors through social media messaging applications, e.g. WhatsApp [53, 63], or online platforms [62]. Students valued these online study groups because they enabled them to discuss details of cases, as well as post and respond to clinical questions [53, 62, 63]. Group members valued the opportunity to immediately resolve complex cases when separated geographically [53], and in one case felt encouraged to participate when able to anonymously post queries without fear of judgement [63].

Students’ use of internet-linked devices to ask questions and discuss clinical issues in a group was also reported from students in institutions that were not formally directing mLearning. Here participants reported value in both large and open, and selective and closed social media-facilitated groups [53]. Views were also shared on structured co-operative peer assessment approaches. Students in one study were positive about the exchanges enabled by use of open source software for the production of sharable ePortfolios [27]. Here students could make comments on each others’ learning portfolios and could rate each others’ comments. In two studies, junior and more experienced students were asked to do pair work at a distance using Skype-enabled devices. Here, both work on case studies in real time [65] and peer-evaluation of clinical skills [53] were described favourably – in one study more so by the junior students [65].

Explanatory or contextual detail of these experiences of co-operative mLearning was described in two studies. In one, students reported being able to form social groups with a sense of cohesion and belonging through learning with smartphones when in remote settings [53]. In another, some felt such phones could ‘keep you connected’ when on clinical assignments [39].

In terms of interactions with teaching staff, students reported particularly valuing being able to instantly contact their supervisors remotely on a variety of topics ranging from discussion of patients’ symptoms [63] to queries on workplace schedules [36, 44, 46, 66] through text and chat. Some reported also being provided with emotional support through these means [46, 53, 62, 66]. There were reports, on the other hand, that some clinicians preferred to use alternative means than smartphones for their teaching work, including PCs for assessments [40], or paper [56].

## Organising learning using mobile devices

Students and staff described using mobile devices to help them organise their learning, for example to access information on learning activities when in a clinical setting [27, 34, 45, 47, 62, 66]. Value was placed on devices that could help organise demands from both clinical practice and academic institutions, with mention of PDAs for tracking the completion of both academic and clinical tasks [25] and for accessing lesson timetables [45]. During and away from clinical practice, students also described how device portability allowed them to better organise their working time, for example using blocks of time in between patients or when waiting for senior staff for learning [2, 31, 40, 45, 47, 51, 62]. Students made favourable comparisons with mobile devices over ‘cumbersome’ text books [60].

## Reflective learning for clinical practice

Students reported using mobile devices to guide their own learning within a clinical context through reflection on learning materials. They described repeatedly viewing medical texts [12, 25, 37, 39, 45, 64] to consolidate their learning or update their knowledge [12, 28, 35]. Both learners and those teaching them described how these activities enabled students to prepare immediately before encounters or achieve more immediate, or more long-lasting insights into a clinical issue [10, 38, 40, 57]. The potential mechanisms for enhanced learning (e.g. authentic problem solving, increased access to immediately relevant or difficult to access clinical cases) were described by both students and staff [10, 45, 53].

Some reported, though, that they were ‘too busy’ to incorporate devices into their learning activities during practice [25, 39, 58, 60, 62]. For example some commented they had little time to input clinical activities for the purposes of reflection [39]; while others found they had insufficient time to view online videos of lectures [62].

# **Mobile Learning Processes**

This section presents findings about study participants’ reflections upon mobile learning as a whole process. It contains sub-themes that represent views on how students learning might be changing and about the process of learning how to learn with mobile devices.

## Changes in pedagogy and learning

Some students reported a shift in the nature of tutor-student relations, in that they felt more able to generate discussion with senior colleagues because of easier access to information [10, 48, 56]. Tutors described a process of learning alongside their students [46, 49]. Enthusiasm for mLearning approaches, however, was far from universal, with some students articulating a preference for traditional pedagogical approaches [25, 34, 38, 40, 52, 56, 61, 65, 66]. Emphasis here was given to the value of paper-based learning [25, 49, 61] and face-to-face communication [52, 66], with some tutors expressing reluctance to invest time integrating a mobile device into their daily schedule [30].

## Learning to mLearn

Some students and lecturers expressed frustration and impatience in the process of learning how to use a device [41, 47, 61]. Even students who were perceived by study authors as technologically capable [34] were seen only to have knowledge of a small number of apps or platforms that they used for their own needs. Study participants described a reliance on others, in particular peers and friends, but also clinical tutors [33, 34, 37, 53] for learning how to use a device. Students in several studies described how they had become more comfortable with mobile devices over time [27, 40, 49, 64], with some claiming that trial and error were key [30, 37, 68]. Technologically more competent users, however, also reported the need for support and repeat training to help them gain sufficient device familiarity [39, 59]. Others reported subtle changes in their learning behaviours over time [37, 61], e.g. ensuring they always recharged their device battery so it would be ready for work use [37].

In a number of studies, either students or tutors expressed insights into the need for evaluation of mLearning opportunities. Uncertainty was voiced over the trustworthiness or reliability of information being distributed through mLearning apps or websites [28, 35, 36, 47, 54, 59, 67]. Students described seeking recommendations for apps from their peers or instructors [36].

# **The institutional implementation of mLearning**

Study participants reported challenges with mLearning that had little to do with interactions between students, devices and their contents, patients and tutors. Here what was implicated were insufficient institutional structures and resources, a lack of device-focused training and support, and limited planning and leadership of mLearning programmes.

## Institutional infrastructure and resources

The importance of network connectivity was emphasised by both tutors and students [2, 42, 45, 60]. The availability of this particular resource was variable as participants in several studies described how Wifi hotspots were unavailable in the educational institutions or hospitals they were working in [35, 40, 61, 67, 68], or that access could be inconsistent, slow, or otherwise poor [38, 40, 42, 51], in particular for video streaming. Both tutors and students reported delays to the roll-out of mLearning programmes due to insufficient Wifi [30, 35, 47]. The use of mobile data plans in place of Wifi was viewed as problematic in some studies [35, 39, 68] due to the associated costs for students.

When students were loaned mobile devices as part of the study, it was noted that such devices were either old, ill-suited to mobile technology [33, 45] or did not work [51, 61]. These studies indicated that institutions may not have the resources to provide students with mobile devices; in one such study, students complained about sharing devices [29].

## mLearning training and technical support

Students expressed a degree of satisfaction with training on how to use their mobile device for the purposes of learning in a small number of studies [39, 40, 55, 59, 68]. In a few instances, they reported feeling empowered enough to incorporate mLearning techniques into their daily activities [55, 59, 68]. Features of these training formats were that they were delivered in an experiential format [49, 59] or were provided throughout the course [41, 60]. Where there was no training, learners reported having insufficient time to familiarise themselves with the device and so to use it for learning [25]. Students and staff in several studies, however, described still being unfamiliar with device functions despite training [34, 38, 41, 58, 60, 68].

Assistance after training or technical support was identified as lacking, for example learners forgot the functions covered during orientation [34, 41, 58, 60, 68]. Technical support was described by teaching staff [45] and learners [41] as helpful during learning activities and later as part of a routine [47]. Students without such a service, described feeling helpless or even panicking. Local technical support, however was described by some learners as fragmented or uncoordinated [33], with a prolonged turn-around time [68]. In one study, a situation was described where support staff might offer software maintenance yet be unable to resolve hardware issues, resulting in users being directed to the device manufacturer [33].

## mLearning leadership and policy

The use of mLearning strategies did not always appear to have been planned with course content or pedagogy in mind, or with consideration of the attributes required by teaching staff. In terms of learning structure, participants in one study complained of poor timing [62]. In one study, students noted that an mLearning module providing sleep education had been introduced during rotation, when medical students often are too busy to view course content online [62]. They, also, reported device content that was not congruent with the rest of their curriculum or reflective of their practice [40, 41, 62]. Students in several studies reported they were offered little guidance on how to integrate mobile devices into their learning activities [34, 38]. Others noted their clinical instructors’ lack of device knowledge [37, 38, 40, 56].

There were, also, several references to disapproval for device-use among supervising staff in clinical settings, resulting in students being hesitant to use the device openly [12, 40, 48, 53, 54, 56, 59]. In one study students reported active discouragement from some supervisors [12]. A range of proposals for guiding practice were made across the studies. In one, senior teaching staff emphasised the importance of clear policies and of improving staff awareness about the value of portable devices [45]. Tutors in another recommended the development of ‘ground rules’ ‘to legitimise… use and ensure entrustability’ (p.130) [48]. The need for ‘behavioural etiquette’, or ‘appropriate, informal codes of conduct’ for digital communication was found to be a consistent discussion theme in one study where students, administrative professionals and tutors all participated [34, 53].
